# Effects of Bile on Pathogenic *Vibrio*, *Aeromonas*, and *Clostridioides* spp. Toxin Effector Domains

**DOI:** 10.3390/biom15111539

**Published:** 2025-11-01

**Authors:** Jaylen E. Taylor, David B. Heisler, Eshan Choudhary, Elena Kudryashova, Dmitri S. Kudryashov

**Affiliations:** 1 Department of Chemistry and Biochemistry, The Ohio State University, Columbus, OH 43210, USA; taylor.3852@osu.edu (J.E.T.); heislerd@duq.edu (D.B.H.); echoudhary@neomed.edu (E.C.); kudryashova.1@osu.edu (E.K.); 2 The Ohio State Biochemistry Program, The Ohio State University, Columbus, OH 43210, USA

**Keywords:** bile, toxin inactivation, innate immunity, *Aeromonas hydrophila*, *Vibrio cholerae*

## Abstract

Bile acids, the primary components of bile, are cholesterol-derived molecules synthesized in the liver and secreted to the small intestine. Besides their primary digestive roles, bile acids have antimicrobial properties and serve as an environmental cue for intestinal pathogens, modulating the expression of virulence factors, e.g., toxins and effector proteins. Whereas timely recognition and neutralization of pathogenic toxin effectors by the host is critical, our understanding of the effects of bile on their structure and function is limited. In this work, we found that bile effectively protected cultured IEC-18 enterocytes from the mixture of *Aeromonas hydrophila* secreted toxins, containing hemolysin, aerolysin, and RtxA (MARTX). To explore whether these effects have broad specificity, we employed biochemical and biophysical techniques to test the in vitro effects of bile and bile acids on several effector domains of MARTX and VgrG toxins from *Vibrio cholerae* and *Aeromonas hydrophila*, and catalytic domains of TcdA and TcdB toxins from *Clostridioides difficile*. Bile compromised the structural integrity of the tested effectors to various degrees in a protein charge-dependent manner. Bile and bile acids promoted exposure of hydrophobic residues and the unfolding of most, but not all, of the tested effectors, facilitating their precipitation and cleavage by chymotrypsin. Bile also inhibited specific activities of the tested effector enzymes, partially due to imposed oxidation of their catalytic residues. To summarize, this work validated bile as a non-proteinaceous factor of innate immunity, capable of compromising the structural integrity and function of the effector domains of various bacterial toxins.

## 1. Introduction

Proteinaceous effector proteins and toxins are major factors differentiating commensal and pathogenic bacteria, enabling the latter to compromise the host’s defense mechanisms, causing illness or death. Therefore, the timely and effective neutralization of bacterial toxins is an imperative task for the host’s immune system upon pathogenic bacterial infection. Currently, only α-defensins are recognized as human innate immune factors capable of neutralizing a broad range of bacterial toxins and effector domains [1,2,3,4,5,6,7]. α-Defensins promote the unfolding of bacterial effectors by taking advantage of their high structural plasticity and low thermodynamic stability [4,8]. These characteristics are essential for either a flawless transition from the water-soluble to membrane-integrated conformation (for pore-forming toxins) [9,10] or for passing through narrow pores in the unfolded state during cytoplasmic delivery [11]. The activity-defining properties of defensins are their amphipathicity, the presence of cationic Lys or Arg residues, structure stabilization by disulfide bonds, and the ability to form stable non-covalent dimers [4,7,8,12,13,14,15,16]. Since bile acids share several similar properties (e.g., rigid structure and amphipathicity), we hypothesized that they may also share toxin-neutralization mechanisms with defensins.

Bile acids, being the primary components of bile, are diverse, multifaceted cholesterol-derived molecules with amphipathic properties. The synthesis of bile acids begins in the liver, where they are stored in the gall bladder, secreted to the proximal small intestine, and reabsorbed in the distal small intestine via the portal vein. In addition to their primary role in digestion, bile acids contribute to homeostasis and dysbiosis of the gut microbiome [17]. Due to their detergent-like properties and the related ability to disrupt the bacterial membrane, bile acids have antimicrobial properties at physiological concentrations, causing oxidative stress and DNA damage [18,19]**.** Being a characteristic component of the small intestine, bile acids are utilized by bacteria as an environmental factor, triggering specific responses and influencing the colonization and virulence of enteric pathogens, such as *V. cholerae* [20,21,22,23,24,25,26], *V. parahaemolyticus* [27,28,29], *Salmonella typhimurium* [30,31], and pathogenic strains of *Escherichia coli* [32,33,34,35]. The combination of amphipathic, surface-active properties of bile acid and the thermodynamic plasticity of many bacterial effectors [11] prognosticates the possible destructive effects of bile on the effectors. Indeed, a precedent study has demonstrated a neutralizing interaction between bile acids and *C. difficile* TcdB toxin, which undergoes structural changes in the presence of bile acids, preventing its binding to target receptors on the host cell’s surface [36]. Yet, the interaction between bile acids and bacterial effectors and toxins has not been systemically explored.

To address this gap in knowledge, we characterized the effects of whole bile and selected bile acids on the properties of representative bacterial effector domains: *C. difficile* TcdA and TcdB glycosyl transferase domains (GTD), actin-binding domain of *S. typhimurium* SipA (SipA_C_), effector domains of *V. cholerae* VopF and VgrG1, and domains of multifunctional autoprocessing repeats-in-toxin (MARTX) from *V. cholerae* and *A. hydrophila*. The majority of the tested proteins showed signs of destabilization in the presence of bile, as revealed by several independent experimental approaches. Each of the effects was observed for the majority but not all the effectors, emphasizing the complexity of the bile-protein interactions. Furthermore, bile and bile acids notably reduced the specific enzymatic activities of the tested effectors. Overall, the current study demonstrates protective effects of bile against a range of bacterial effectors, pointing towards its important role as a factor of innate immunity.

## 2. Materials and Methods

### 2.1. Protein Expression and Purification

Actin was purified from rabbit acetone powder (Pel-Freeze Biologicals, Rodgers, AR, USA) as previously described [37]. Actin was labeled with Oregon Green 488 maleimide (Molecular Probes/Thermo Fisher Scientific, Waltham, MA, USA) and pyrene iodoacetamide (Thermo Fisher Scientific, Waltham, MA, USA) at Cys374 as described [38]. Unlabeled and labeled actins were purified by size exclusion chromatography (HiPrep 26/30 Sephacryl S-200 HR, Cytiva/GE Healthcare, Marlborough, MA, USA) and stored on ice in G-buffer (2 mM Tris-HCl, pH 8, 0.2 mM CaCl_2_, 0.2 mM ATP, 0.5 mM β-mercaptoethanol [β-ME]).

*V. cholerae* and *A. hydrophila* MARTX effector domain constructs, *Salmonella* SipA C-terminal domain (SipA_C_), and *V. cholerae* VopF (residues 129-530) were expressed in *E. coli* BL21(DE3)pLysS cells (Agilent Technologies, Santa Clara, CA, USA), and purified as described [11,39,40]. *C. difficile* GTD constructs from TcdA (amino acids 1-542) and TcdB (amino acids 1-543) were expressed in *Bacilus megaterium* as previously described [41,42]. Human plastin 2 (PLS2) and cofilin 2 (CFL2) were expressed in *E. coli* BL21 CodonPlus(DE3)-RP and *E. coli* BL21(DE3) cells (Agilent Technologies, Santa Clara, CA, and MilliporeSigma, St. Louis, MO, USA), respectively, and purified as described [39,43]. Recombinant human plasma gelsolin (GSN) was expressed in *E. coli* BL21(DE3)pLysS cells and purified by ion exchange chromatography using DEAE followed by DE52 column (Cytiva, Marlborough, MA, USA).

### 2.2. Bile Salt Preparation

Dried, unfractionated bovine bile (ox gall powder; Sigma Aldrich, St. Louis, MO, USA) was dissolved in deionized water and used within 1 day. Solutions of purified bile acids, sodium deoxycholate, and sodium taurocholate (Sigma Aldrich, St. Louis, MO, USA) were prepared fresh and used within 2 days. The experiments were conducted either in phosphate-buffer saline (PBS, pH 7.4; Sigma Aldrich, St. Louis, MO, USA), sodium phosphate buffer, or in Tris-based buffer as specified below. It was verified that the pH of the final solutions was not substantially affected by bile and bile acids.

### 2.3. Cell Culture

IEC-18 rat small intestine epithelial cells (CVCL_0342) were grown in Dulbecco’s modified Eagle medium (DMEM; Sigma Aldrich, St. Louis, MO, USA) supplemented with 10% fetal bovine serum (FBS; Thermo Fisher Scientific, Waltham, MA, USA), penicillin-streptomycin and GlutaMAX (Thermo Fisher Scientific, Waltham, MA, USA) at 37 °C in a humidified incubator under 5% CO_2_.

### 2.4. Cytotoxicity Assays

Secreted membrane-damaging toxins were obtained by growing *A. hydrophila* (Chester) Stanier (ATCC 7966; Manassas, VA, USA) in LB media overnight and separating the media from the bacteria by centrifuging at 4000× *g* for 10 min. The supernatant was collected, filter-sterilized, and lipopolysaccharide (LPS) was removed using Pierce high-capacity endotoxin removal resin (Thermo Fisher Scientific, Waltham, MA, USA), as confirmed by Pierce chromogenic endotoxin quantification kit (Thermo Fisher Scientific, Waltham, MA, USA). Samples with LPS concentration below 0.5 EU/mL were considered LPS-free. At this stage, the *A. hydrophila* supernatant was aliquoted and frozen. Only freshly thawed aliquots were used for cytotoxicity assays to ensure the reproducibility of the experimental replicates. Within the experimental day, *A. hydrophila* media sample was melted in a water bath at 37 °C and split into aliquots that were incubated in the absence or presence of 0.1% bile for 45, 5, or approximately 1 min or less, the latter representing the minimum time required for thorough mixing of the media and bile solution just before application to IEC-18 cells.

IEC-18 cells were seeded in a 96-well plate at 70% confluency and allowed to grow overnight to 100% confluency. *A. hydrophilia* supernatant supplemented with 0.1% bile was diluted to 10% *v*/*v* in complete growth medium, then applied 1:1 to 100% confluent cells. The final concentration of *A. hydrophila* supernatant applied to cells was 5% *v*/*v*. Over the course of 5 h, membrane integrity of IEC-18 cells was assessed by phase contrast and fluorescence microscopy after treating cells with a 1.25 μg/mL solution of the membrane-impermeable dye erythrosin B (Thermo Fisher Scientific, Waltham, MA, USA) in PBS (Sigma Aldrich, St. Louis, MO, USA) for 1 min. Experiments were performed in triplicate. Micrographs were obtained using a Nikon Ellipse TiE microscope (Nikon Instruments Inc., Melville, NY, USA) with 20× objective. Mean erythrosin B fluorescence and percentage of area cell coverage were calculated using ImageJ/FIJI software (Verision 2.1.0) [44] from fluorescence and phase contrast images, respectively.

### 2.5. Liquid Chromatography Coupled Tandem Mass Spectrometry (LC-MS/MS)

Prior to LC-MS/MS analysis, *A. hydrophila* supernatant samples were flash-frozen in liquid nitrogen and stored at −80 °C before use. Samples were thawed and reduced by the addition of 3.24 mM dithiothreitol (DTT) at 65 °C for 15 min, followed by alkylation with 8.11 mM iodoacetamide (IAA) in the dark for 30 min. Proteins were acidified to 1.2% H_3_PO_4_, and S-Trap binding buffer (90% methanol, 50 mM triethylammonium bicarbonate (TEAB)) was added in a 1:6 sample/S-Trap binding buffer (*v*/*v*) ratio, before loading them onto the S-Trap column by centrifugation at 4000× *g* for 1 min. The sample was washed three times with S-Trap binding buffer, followed by centrifugation with no solution to remove any excess methanol. S-Trap columns were transferred to a clean tube, and sequencing-grade trypsin (Promega, Madison, WI) was added to the sample in a 1:100 trypsin/protein (*w/w*) ratio dissolved in 50 mM TEAB. The proteomics sample was incubated overnight at 37 °C. Peptides were eluted sequentially using (1) 50 mM TEAB, (2) 0.2% formic acid in H_2_O, and 3) 0.2% formic acid in 50% acetonitrile.

The samples were analyzed on a Bruker TIMS ToF Pro (Bruker Daltonics, Billerica, MA, USA) coupled to a Nano Elute 2 Liquid Chromatography system (Bruker Daltonics, Billerica, MA, USA) in the Mass Spectrometry & Proteomics Campus Chemical Instrument Center at The Ohio State University. 2 µL of peptides in buffer A (0.1% formic acid in water) were separated on an Aurora Ultimate C18 column (25 cm × 75 µm ID) at 50 °C. Buffer B (0.1% formic acid in acetonitrile) was increased to 17% over 60 min, then increased to 25% in 30 min, and lastly, increased to 37% in 10 min. Buffer B was then increased to 80% over 10 min and held at 80% for 10 more minutes. The TIMS ToF Pro was set to ddaPASEF (data-dependent acquisition Parallel Accumulation Serial Fragmentation) mode, scanning from *m*/*z* = 100 to *m*/*z* = 1700 with a 1/K_0_ start of 0.60 V·s/cm^2^ and ending at 1/K_0_ 1.60 V·s/cm^2^ with a 100 ms ramp time. For tandem MS, a total of 10 PASEF ramps were performed, followed by elution and collision-induced dissociation (CID) with nitrogen at a stepped collision energy based on precursor mobility.

The samples were searched using MSFragger within the Fragpipe Graphical User Interface [45]. A precursor mass tolerance of ±15 ppm and a fragment mass tolerance of ±15 ppm were used. For all samples, carbamidomethylation of cysteine (+57.021 Da) was set as a static modification, and methionine oxidation (+15.995) and N-terminal acetylation (+42.011) were set as variable modifications. MSBooster [46] was enabled to predict spectra and retention times using the Prosit 2019 model [47]. Percolator was used for PSM validation, and ProteinProphet was enabled for protein summarization to a 1% False Discovery Rate (FDR). Spectral counts were then used to determine abundance of various bacterial toxins.

### 2.6. Collisional Quenching

The solution accessibility of native Trp residues in the analyzed toxins was measured by collisional quenching of their intrinsic Trp fluorescence upon titration by acrylamide (0–750 mM, in 4 μL aliquots) in the presence and absence of reconstituted bovine bile and bile acids (deoxycholate, DOCh, and taurocholate, TCh). Trp fluorescence was excited at 295 nm, and emission was recorded at 327 nm using a PTI QuantaMaster spectrofluorometer (Horiba Instruments, Piscataway, NJ, USA). Intrinsic fluorescence of bile alone (final concentration 0.1% *w*/*v*) at each concentration of acrylamide was subtracted from the experiment in the presence of bile. DOCh and TCh did not demonstrate intrinsic fluorescence, and the correction was not necessary.

The ratio of the initial fluorescence intensity (*F_o_*) to the fluorescence at a given concentration of acrylamide quencher (*F*) was plotted as a function of the acrylamide concentration. The experimental data was fit using the Stern–Volmer equation:

(1) $$\frac{F_{o}}{F}=K_{sv}+1$$

where *K_sv_* is the Stern–Volmer coefficient determined from the slope of the linear fit.

Published and AlphaFold2-predicted structures [48,49,50,51] were analyzed in PyMOL (The PyMOL Molecular Graphics System, Version 2.5.2). Trp residues are labeled red, and Arg657 of SipA_C_ in proximity to the only Trp is labeled yellow (Figure S2).

### 2.7. Guanidinium Chloride Induced Denaturation

Chemical denaturation of MARTX toxin effector domains (4 μM final concentration) in 20 mM Tris-HCl, pH 7.5, 150 mM NaCl) was monitored via changes in the maximum fluorescence of Trp residues upon their solution exposure in the presence of guanidinium hydrochloride (GdmCl) in the absence and presence of bile. Measurements were conducted in a 384-well low-volume non-binding black polystyrene plates (Corning Inc., Corning, NY, USA). Intrinsic fluorescence of Trp residues was measured using an Infinite M1000 Pro plate reader (Tecan US Inc., Morrisville, NC, USA), at an excitation of 295 nm at room temperature (25 °C). The experiments were performed in triplicate. The maximum wavelength of the emission peaks from the averaged spectra was plotted with its corresponding GdmCl concentration.

### 2.8. Circular Dichroism (CD) Spectroscopy

Far-UV CD spectra were obtained using the JASCO J-1500 CD instrument (JASCO analytical instruments, Oklahoma City, OK, USA) equipped with a Peltier temperature controller and quartz cuvettes of 0.1 cm path length. CD, dynode voltage, and absorbance of 0.25 mg×mL^−1^ samples in 50 mM sodium phosphate buffer, pH 7.4, were recorded at a scan speed of 100 nm×min^−1^, using 1 nm bandwidths. Entire far-UV spectra were recorded every 2 °C during thermal ramping (1 °C/min) from 20 °C to 94 °C with a wait time of 5 s before measurements at each temperature. Far-UV CD signals were expressed as mean residue molar ellipticity, [*θ*]:

(2) $$\left[\theta \right]=\frac{\theta \times 100\times M}{C\times l\times n}$$

where *θ* is the ellipticity (millidegrees), * M* is the molecular weight (kDa), *C* is the protein concentration (mg×mL^−1^), *l* is the optical path (cm), *n* is the number of amino acid residues.

### 2.9. Reconstruction of the Secondary Structure Composition

The BeStSel software (Version 1.3.230210) [52] was used to predict the protein secondary structure content from the far-UV CD spectra. The predicted percent secondary structure content of α-helices, antiparallel β-sheets, and parallel β-sheets was plotted against the thermal ramp from 20 °C to 94 °C and analyzed for changes in the presence and absence of bile.

### 2.10. High-Speed Ultracentrifugation

The selected toxins and human proteins (5 μM final concentration) were incubated for 45 min at 20 °C or 37 °C in a thermocycler in the presence and absence of 0.25% *w*/*v* whole bile or DOCh. Following the incubation, the proteins were centrifuged at 300,000×g using a Beckman Optima TLX ultracentrifuge (Beckman Coulter, Brea, CA, USA) in a TLA-100 rotor for 30 min at 20 °C or 37 °C. The supernatant and pellet fractions were separated, and protein was visualized on 12% SDS-PAGE gels. Precipitated protein (fraction pelleted) was calculated by densitometry of the Coomassie-stained gel bands using ImageJ/Fiji (Verision 2.1.0) [44] and normalizing the protein level in the pellet to the total protein present in the supernatant and pellet fractions combined for each experimental condition.

### 2.11. Native Polyacrylamide Gel Electrophoresis (Native PAGE)

Aggregation of bacterial toxin effectors in the presence of bile and DOCh was assessed by native PAGE. The selected toxins were incubated in the presence and absence of 0.1 or 0.25% *w*/*v* bile or DOCh at 20 °C or 37 °C. Samples were supplemented with 5× native sample buffer (156.25 mM Tris-HCl, pH 6.8, 31.25% glycerol, 0.04% bromophenol blue) and visualized on 7.5% native PAGE gels under reducing (Figure S7A, top) and non-reducing (Figure S7A, bottom) conditions.

### 2.12. Cysteine Protease Domain (CPD) Cleavage Activity Assays

The cysteine protease activity of CPD_MARTX_ was assessed using ABH-CPD_MARTX_ or ACD-RID-ABH-CPD_MARTX_ (also referred to as 4d_MARTX_) as substrates. The cleavage reactions were initiated by adding 50 μM of inositol-hexakisphosphate (phytic acid, IP_6_; Sigma Aldrich, St. Louis, MO, USA) in 20 mM Tris-HCl, pH 7.5, 150 mM NaCl to 3 μM of ABH-CPD_MARTX_ or 4d_MARTX_. The reactions were incubated at 37 °C and inhibited at the designated time points by the addition of 1 mM N-ethylmaleimide (NEM). The samples were mixed with equal volumes of a 2× non-reducing sample buffer (62.5 mM Tris-HCl, pH 6.8, 25% glycerol, 2% sodium dodecyl sulfate (SDS), 0.0016% bromophenol blue) and visualized on 12% SDS-PAGE gels. For whole bile and bile salt conditions (DOCh, TCh), toxins were incubated at 37 °C with 0.1% *w*/*v* either whole bile, DOCh, or TCh for 1 h before initiating the cleavage reaction by adding IP_6_. For reactions in the presence of DTT or ethylenediaminetetraacetic acid (EDTA), toxins were incubated at 37 °C in 10 mM DTT or 20 mM EDTA for 1 h before the reactions were initiated with IP_6_. The effects of bile on the ABH-CPD cleavage were tested with minor variations five times with highly similar outcomes; the effects of DOCh and TCh were tested three times, and the effects of EDTA two times with a reproducible outcome. Gels were stained with Coomassie brilliant blue and quantified by densitometry using ImageJ/Fiji software (Verision 2.1.0). Accumulation of cleavage products and depletion of ABH-CPD_MARTX_ upon activation of CPD activity were analyzed and plotted as a function of time. Fraction of control was calculated by dividing the density of the ABH-CPD_MARTX_ protein band at each time point by the density of the ABH-CPD_MARTX_ band at t = 0 min for each experimental condition. Quantification of the accumulation of the cleavage products (ABH_MARTX_ and CPD_MARTX_) was adjusted to the difference in the molecular weights of ABH-CPD_MARTX_ (60.9 kDa), ABH_MARTX_ (38.8 kDa), and CPD_MARTX_ (24.2 KDa). Accumulation of the individual cleavage products of *V. cholerae* 4d_MARTX_ due to CPD cleavage was quantified and adjusted in the same manner (4d_MARTX_, 185.3 kDa; RID_MARTX_, 74.7 kDa; ACD_MARTX_, 54.0 kDa; ABH_MARTX,_ 38.8 kDa; CPD_MARTX_, 24.2 kDa).

### 2.13. Limited Proteolysis

Bacterial effectors and recombinant human proteins were proteolytically cleaved by trypsin and chymotrypsin in the presence and absence of 0.1% *w*/*v* reconstituted whole bovine bile. A 3-μM sample of each of the tested proteins was incubated with trypsin (Worthington Biochemical, Lakewood, NJ, USA) and chymotrypsin (alpha-chymotrypsin; Worthington Biochemical, Lakewood, NJ, USA) at 1:2500 and 1:2000 molar ratios, respectively, over the course of 30 min. The cleavage was stopped at the designated time points by transferring 20-µL aliquots from the reactions to Eppendorf tubes containing a final concentration of 1 mM phenylmethylsulfonyl fluoride (PMSF). Samples were mixed with an equal volume of 2× reducing SDS-sample buffer (62.5 mM Tris-HCl, pH 6.8, 25% glycerol, 2% sodium dodecyl sulfate (SDS), 0.0016% bromophenol blue, 7.15 mM β-ME) and analyzed by SDS-PAGE in a similar manner to the CPD cleavage assays.

### 2.14. TMR-5-Maleimide and DCP-Rho1 Labeling Assays

*V. cholerae* ABH-CPD_MARTX_ (5 μM final concentration) was incubated at 37 °C for 30 min in the presence and absence of 0.1% *w*/*v* bile in 20 mM Tris-HCl, pH 7.5, 150 mM NaCl. After incubation, bile was removed by passing the solution via a NAP-5 gravity size-exclusion column (GE Healthcare, Chicago, IL, USA). The protein was recovered, and CPD cleavage was activated by the addition of 250 μM IP_6_. The redox state of the catalytic cysteine of CPD was assessed by labeling with a thiol-specific tetramethylrhodamine-5-maleimide (TMR) probe or DCP-Rho1, a selective sulfenic acid reactive rhodamine probe (Cayman Chemical, Ann Arbor, MI, USA), for 1 h at room temperature, followed by resolving on non-reducing 12% SDS-PAGE gels. The gels were exposed to 365 nm UV light to detect and image TMR or DCP-Rho1 fluorescence of the labeled proteins, then stained with Coomassie blue R-250 to detect all proteins. Fold change of fluorescently labeled ABH-CPD_MARTX_ relative to untreated sample was calculated from the fluorescence intensity of the protein bands.

### 2.15. ACD_MARTX_ Actin Crosslinking Activity Assays

Actin crosslinking by ACD_MARTX_ was performed as previously described [53] with some modifications. 10 µM G-actin in G-buffer (5 mM Tris-HCl, pH 8.0, 0.2 mM ATP, 0.2 mM CaCl_2_, 1 mM DTT) was supplemented with 1.2 molar excess of latrunculin B (LatB), to prevent polymerization, and 2 mM MgCl_2_, to support the catalytic activity of ACD [54]. The reaction was initiated by the addition of ACD at 1 nM final concentration in the absence or presence of various concentrations of whole bile, added to ACD_MARTX_ immediately prior to the reaction initiation. All reactions were carried out at 25 °C. Aliquots were extracted at the indicated time points and quenched by mixing with 4× SDS-PAGE sample buffer (125 mM Tris-HCl, pH 6.8, 50% glycerol, 4% SDS, 0.03% bromophenol blue, 1.43 mM β-ME). For testing the reversibility of ACD inactivation, ACD at high concentration (5 µM) supplemented with 2 mM MgCl_2_ and 0.2 mM ATP was pre-incubated with the desired concentrations of bile acids. After 5 min of incubation at 25 °C, the enzyme was diluted with the reaction buffer (50 mM Tris-HCl, pH 8.0, 2 mM MgCl_2_, 0.2 mM ATP) devoid of bile acids and further diluted upon its addition to the actin/LatB reaction solution to the final concentration of 1 nM, attaining 5000-fold dilution of ACD and bile acids from their initial concentrations. The crosslinking reaction was quenched at designated time points as specified above. To validate that ACD was inhibited by bile before the dilution, 5 µM of ACD_MARTX_ was incubated in the presence and absence of 0.4% *w*/*v* bile and diluted four-fold by a buffer containing G-actin and low Mg^2+^ concentration to obtain the following final concentrations of the reactants in the experimental mixture: 5 µM actin, 1.25 µM ACD_MARTX_, 0.1% bile, and 20 or 50 µM MgCl_2_. The reaction was allowed to proceed for 1 min at room temperature before it was quenched with 4× SDS-PAGE sample buffer. The experiment was conducted in triplicate. The samples were resolved on 9% SDS-PAGE gels and stained with Coomassie brilliant blue R-250. Densitometry analysis was performed using ImageJ/Fiji (Verision 2.1.0) [44], and rates of crosslinking were expressed in µmole of crosslinked bonds formed per min.

### 2.16. VopF-Mediated Actin Polymerization

Bulk pyrenyl-actin polymerization assays were performed as previously described [38]. Briefly, 300 nM VopF was diluted in the reaction buffer (10 mM MOPS pH 8, 0.2 mM ATP, 0.5 mM DTT) to 15 nM and combined with 2.5 µM G-actin (5% pyrene-labeled). Pyrene fluorescence was excited at 365 nm and monitored at 407 nm for 1 min prior to switching G-actin from the Ca^2+^-ATP- to the Mg^2+^-ATP-bound state with the addition of 1/15th of the reaction volume of 15× switch buffer (150 mM MOPS, pH 7, 3 mM ATP, 7.5 mM DTT, 4.5 mM EGTA, 1.5 mM MgCl_2_). After 90 s, actin polymerization was initiated by adding 1/3rd of the reaction volume of 3× initiation buffer (30 mM MOPS, pH 7, 0.6 mM ATP, 1.5 mM DTT, 3 mM MgCl_2_, 150 mM KCl), and fluorescence was monitored using Infinite M1000 Pro plate reader (Tecan US Inc., Morrisville, NC, USA) until the polymerization curves reached a plateau. Spontaneous actin polymerization was monitored in the absence of VopF, while the VopF-assisted polymerization—at a final concentration of 5 nM VopF. Changes in fluorescence traces were normalized to the total change in fluorescence due to the auto-fluorescence of the bile salt solutions, which increased linearly with bile concentration.

### 2.17. Statistical Analysis

Experimental data for each experiment is presented as mean ± standard deviation (SD) or standard error (SE), as specified in the Figure legends. *p*-values were calculated using the two-tailed *t*-test in Microsoft Excel. Cell culture experiments were performed in triplicate, with *p*-values calculated relative to control. Collisional quenching experiments were performed in at least three independent repetitions. GdmCl denaturation experiments were conducted in three independent experiments, measured in triplicate; means of each triplicate experiment were averaged and presented as mean ± SD. Limited proteolysis experiments were conducted as independent replicates with three or more repetitions. High-speed precipitation experiments were conducted as independent replicates; data are plotted as means; error bars represent SE. Actin crosslinking and pyrenyl-actin polymerization experiments were performed as three independent replicates. Correlation analyses of the dependences of *K_SV_*, *θ*_208_, and pelleted fractions versus pI were performed using Prism 10.5.0 (Graph Pad). All graph data are available in the Supplementary Materials (Data S1).

## 3. Results

### 3.1. Bile Inactivates A. hydrophila Exotoxins and Protects Cultured Enterocytes

**Figure 1:**
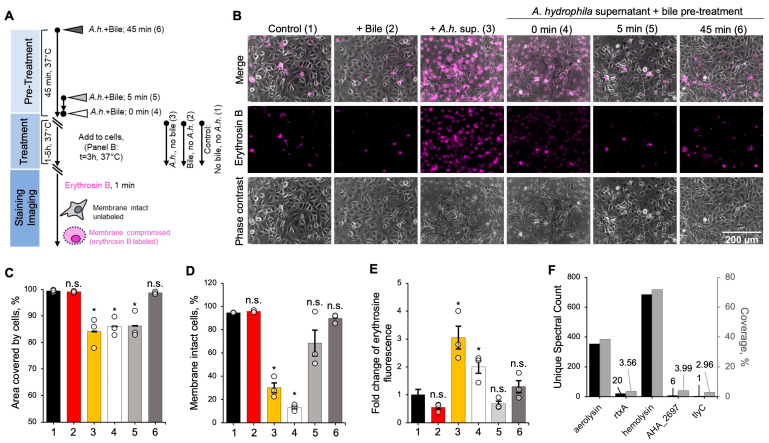
Bile inhibits toxicity of *A. hydrophila* supernatant against IEC-18 cells. (**A**) Experimental workflow of the cytotoxicity assays. (**B**) Micrographs show overlay of phase contrast and erythrosin B fluorescence images of IEC-18 cells treated with *A. hydrophila* supernatant for 3 h (1 and 5 h time points are shown in Figure S1A and S1B, respectively). *A. hydrophila* supernatant was untreated or pre-treated with bile for the indicated amount of time prior to the addition to cells. (**C**–**E**) Cytotoxicity effects were quantified as the percentage of area covered with cells (**C**), the percentage of cells with intact membrane (erythrosin B-negative) for each treatment (**D**), and a fold change in the erythrosin B fluorescence relative to the untreated control (**E**). The data from experiments conducted in triplicates (*n* = 3) are presented as mean ± standard error (SE). *p*-values were determined using two-tailed Student’s *t*-test comparing each group with the untreated control. (1) Untreated control, black; (2) bile, no *A.h.*, red; (3) *A.h.*, no bile, yellow; (4) *A.h.* + bile, 0 min, white; (5) *A.h.* + bile, 5 min, light gray; (6) *A.h.* + bile, 45 min, dark gray. (**F**) Percent coverage (grey bars) and spectral counts (black bars) indicating abundance of membrane-damaging *A. hydrophila* toxins in the harvested supernatant were determined by tandem mass spectrometry (see also Table S1). *, *p* ≤ 0.05; n.s., non-significant.

To assess the ability of bile to neutralize bacterial toxins, we initially focused on the *A. hydrophila* secreted toxins. *A. hydrophila* is an opportunistic human pathogen that secretes several exotoxins, including hemolysins, aerolysin, and MARTX [55,56,57], each capable of causing major cytotoxicity. We treated normal rat small intestine epithelial IEC-18 cells with supernatants from *A. hydrophila* (strain ATCC 7966) for 1–5 h; the supernatants were untreated or pre-treated with 0.1% bile for various intervals of time (0–45 min) (Figure 1A). Within 1 h of application, the control, untreated *A. hydrophila* supernatant caused visible cell shrinkage (Figure S1A). Assessing the cell integrity with a fluorescent membrane-impermeable probe, erythrosin B, revealed compromised membranes as early as 1 h post-treatment (Figure S1A), as judged by the accumulation of the probe within the cell. By 3 h, the effects were even more notable, with obvious changes in cell morphology (Figure 1B), a 15% decrease in cell size, and a 64.6% increase in intracellular erythrosin B fluorescence (Figure 1C–E). At 5 h post-treatment, most cells succumbed to the toxic effects of the *A. hydrophila* supernatant (Figure S1B). In striking contrast, 5-min pre-incubation of the supernatant with bile (0.1% *w*/*v*) prior to addition to cells effectively protected the cells, remarkably reduced cytoplasmic erythrosin B staining, and moderately lessened morphological changes (Figure 1B–E). After 45 min of pre-incubation, the signs of toxicity were almost nullified, to the extent that IEC-18 cells had no changes in morphology or the cell size (as measured by the cell-covered area) (Figure 1B–E). Tandem MS/MS analysis of the *A. hydrophila* supernatant confirmed the presence of several type II secretion system (T2SS) membrane-damaging toxins: hemolysin Ahh1 (AHA_1512), aerolysin AerA (AHA_0438), and RtxA (AHA_1359), in the order of decreasing abundance (Figure 1F, Table S1). These findings confirmed that bile has the capacity to inactivate bacterial toxins, including a broad range of pore-forming toxins secreted to the extracellular space.

### 3.2. Bile and Deoxycholate Promote Exposure of Effectors’ Tryptophan Residues

To understand the mechanism of toxin inactivation by bile, we characterized its effects on effector domains from toxins produced by *A. hydrophila* and other pathogens: glucosyltransferase domains (GTD) of *C. difficile* TcdA and TcdB toxins, an actin-binding domain of *Salmonella typhimurium* T3SS SipA toxin (SipA_C_), and effector domains from *A. hydrophila* and *V. cholerae* MARTX toxins: Rho inactivation domain (RID), actin crosslinking domain (ACD), α/β-hydrolase domain (ABH), and cysteine protease domain (CPD). Such a diverse group of effector domains was selected in an attempt to gain a broader understanding of bile’s effects—not limited to a particular pathogen or a class of toxins.

**Figure 2:**
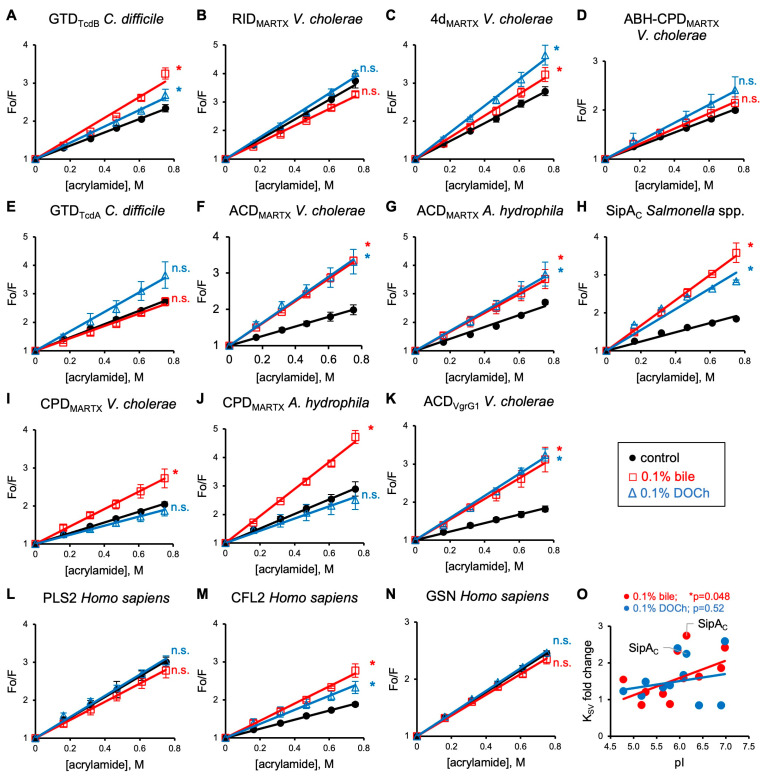
Bile and DOCh expose bacterial effectors’ Trp residues to collisional quenching by acrylamide. (**A**–**N**) Tryptophan fluorescence of bacterial toxins (**A**–**K**) and human proteins (**L**–**N**) was incrementally quenched by acrylamide in the absence or presence of 0.1% reconstituted bile or 0.1% DOCh. The ratio of initial fluorescence intensity to the quenched fluorescence intensity (F_o_/F) was plotted as a function of acrylamide concentration. Stern–Volmer coefficients (K_SV_) were determined from the slope of each quenching experiment using the Stern–Volmer equation as described in the Methods section and compared with the untreated control for statistical significance. Data are presented as mean ± SD from three or more independent experiments. *, *p* ≤ 0.05; n.s., non-significant (two-tailed Student’s *t*-test). (**O**) Fold change of K_SV_ (as compared to the untreated control) was plotted against the isoelectric point (pI) of each tested effector with linear fit lines. *p*-values were determined from correlation analysis. See also Figure S2.

Changes in the structural integrity of proteins can be mapped via solvent accessibility of their Trp residues, a unique protein characteristic that depends on the number of tryptophans and their proximity to the protein surface [58]. We evaluated the effect of bile and deoxycholate (DOCh) on the solvent accessibility of Trp residues of the above effector domains by probing them with acrylamide, an uncharged collisional quencher. For most effectors, Trp quenching was notably increased either by both, whole bile and DOCh (for six effectors; Figure 2A,C,F–H,K) or at least by one of them (for two effectors; Figure 2I,J), as judged by a statistically higher Stern–Volmer coefficient (K_SV_) reflected in a higher slope of the linear fits (Table S2). Of the tested proteins, RID, the fusion of ABH-CPD of *V. cholerae* MARTX toxin, and GTD of *C. difficile* TcdA toxin were resistant to both bile and DOCh, or the observed difference was not statistically significant (Figure 2B,D,E). To test whether the observed bile-mediated quenching potentiation is restricted to bacterial effectors, we also tested recombinant human proteins cofilin 2 (CFL2), plastin 2 (PLS2), and gelsolin (GSN). Whereas the quenching of Trp residues of PLS2 and GSN was not affected by bile and DOCh (Figure 2L,N), the quenching of CFL2 was potentiated by both (Figure 2M). The observed sensitivity of human proteins to bile suggests that the induced structural distortions are not limited to bacterial toxins.

Since many bile acids are negatively charged, we checked whether the scale of collisional quenching may correlate with the effectors’ isoelectric points (pI). Overall, more acidic effectors were more resistant to the bile effects (Figure 2O), with one notable exception, SipA_C_ (Figure 2H). Removal of this obvious outlier from the analysis produced correlation coefficients (r) of 0.637 (*p* = 0.0476) and 0.236 (*p* = 0.5) for bile and DOCh, respectively, reflecting statistically stronger collisional quenching of proteins with less acidic pI in the presence of bile (Figure 2O). This correlation likely reflects electrostatic repulsion between negatively charged effectors and bile acids, many of which are negatively charged. The structural analysis (Figure S2) of SipA_C_, whose Trp fluorescence was quenched at a disproportionally higher level (Figure 2H), showed that the apparent deviation from the rule is due to the immediate proximity of the sole tryptophan of SipA_C_ to an Arg residue (Figure S2I, yellow), whose local positive charge dominates over the protein’s pI in attracting negatively charged bile acids, confirming the overall correlation.

### 3.3. Bile Facilitates Proteolytic Cleavage of Bacterial Effectors at Hydrophobic Residues

**Figure 3:**
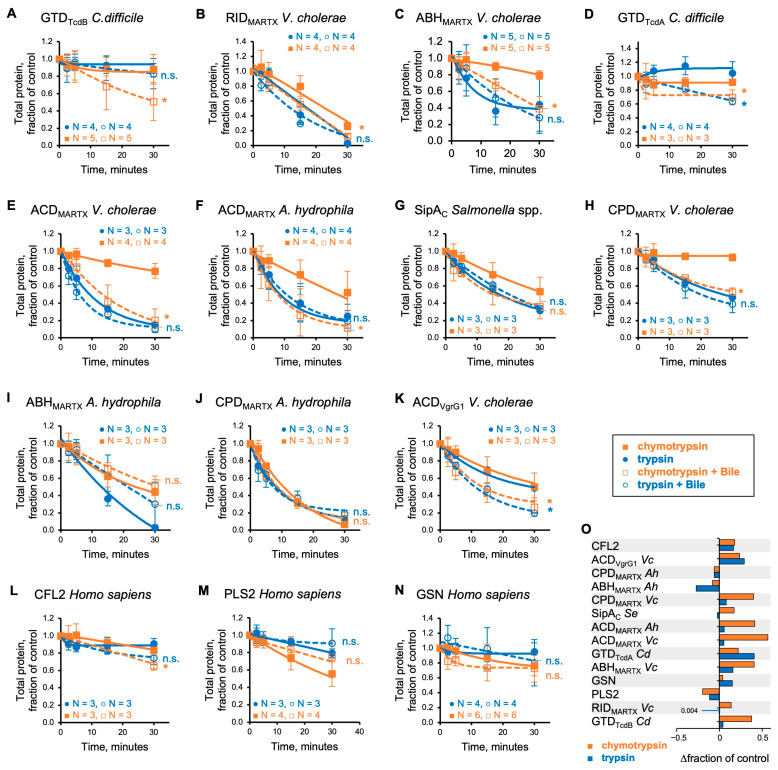
Bile promotes proteolytic cleavage of bacterial effectors at hydrophobic residues. (**A**–**N**) Limited proteolysis of bacterial effectors (**A**–**K**) and human proteins (**L**–**N**) by pancreatic serine proteases, trypsin (blue circles) and chymotrypsin (orange squares), in the presence (dashed lines) and absence (solid lines) of 0.1% *w*/*v* whole bile over 30 min. Data are presented as the average ± SD from three or more independent experiments and a single exponential decay fit. The difference in averaged protein fraction that remained uncleaved at the 30 min time point, compared to the “no bile” controls, was checked for statistical significance using two-tailed Student’s *t*-test (*, *p* ≤ 0.05; n.s, non-significant) as shown in (**A**–**N**) and plotted in (**O**) for trypsin (blue bars) and chymotrypsin (orange bars) cleavage for each protein. See also Figure S3.

To independently validate the detected exposure of hydrophobic residues to solvent by bile, we employed limited proteolysis with pancreatic proteases, trypsin and chymotrypsin, targeting polar Arg/Lys and bulky non-polar Leu/Trp/Tyr/Phe residues, respectively. Of the two types, polar residues are typically exposed at the protein surface, while the hydrophobic ones are hidden but may be exposed by bile, as suggested by the collisional quenching experiments. Indeed, bile notably increased the susceptibility of most of the effectors to chymotrypsin (Figure 3; orange traces) with the exception of *A. hydrophila* ABH_MARTX_ and CPD_MARTX_, and *S. enterica* SipA_C_ (Figure 3G,I,J). Accelerated cleavage was accompanied by the appearance of smaller-sized cleavage products (Figure S3B–O; arrowheads), suggesting the exposure of additional cleavage sites as reported previously for α-defensins [4]. In contrast, the cleavage by trypsin was less affected (Figure 3A–K,O; blue traces). We verified that the changes in proteolysis in the presence of bile were not due to the effects of bile on chymotrypsin (Figure S3A), in agreement with previous findings [59]. Cleavage of the three control human proteins by chymotrypsin was affected only mildly, reaching the significance level only for CFL2 (Figure 3L–N). Interestingly, PLS2 appeared to be mildly protected by bile, but this apparent protection was accompanied by the appearance of additional proteolysis products, pointing to the similar nature of the effects of bile on PLS2 and bacterial effectors.

### 3.4. Chemical Unfolding of Bacterial Effectors by Guanidinium Chloride Is Only Mildly Affected by Bile

**Figure 4:**
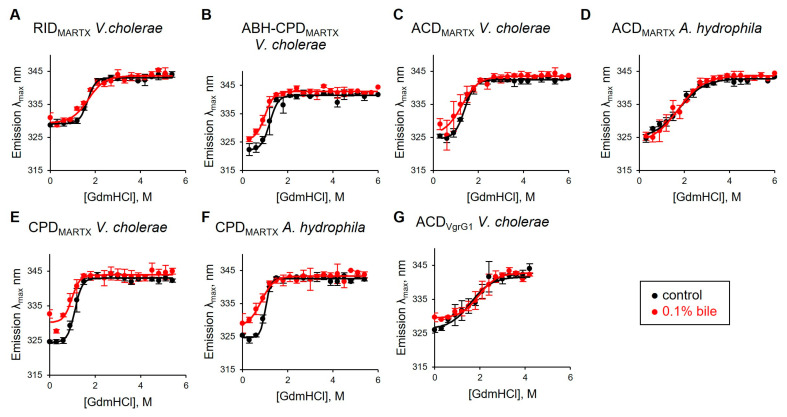
Chemical protein unfolding by GdmCl is unaffected by bile. (**A**–**G**) GdmCl-induced denaturation was conducted on MARTX bacterial effector domains from *V. cholerae* and *A. hydrophila* (**A**–**F**) and *V. cholerae* VgrG1 toxin effector ACD (**G**), in the presence (red) and absence (black) of 0.1% whole bile. Maximum emission wavelengths of native Trp residues were plotted over the GdmCl concentrations at an excitation of 295 nm. Data from three experiments are presented as mean ± SD and shown in Table S2.

For independent assessments of the bile’s effects on protein stability, we evaluated guanidinium chloride (GdmCl)-induced denaturation, focusing on MARTX effector domains from *V. cholerae* and *A. hydrophila* (Figure 4). The intrinsic fluorescence peaks of most of the tested effectors in the absence of GdmCl are red-shifted by ~3 nm in the presence of bile (Figure 4B,C,E–G; Table S2), pointing to exposure of Trp residues to a more polar environment, in agreement with the collisional quenching and proteolysis experiments. Surprisingly, overall chemical denaturation profiles and half-transition points were very similar in the absence and presence of bile, except that some effectors retained the ~3 nm difference in the peak position throughout the titration, whereas melting profiles of others (RID_MARTX_ and ACD_VgrG1_ of *V. cholerae* and ACD_MARTX_ of *A. hydrophila*) overlapped with those of control titrations in the absence of bile. While it appears that bile only marginally affects protein unfolding on a global scale, it is likely that the denaturation-promoting effects of bile are disguised by GdmCl, whose ability to negate the hydrophobic effect may weaken the interaction between bile and the protein core residues.

### 3.5. Effects of Bile and Deoxycholate on the Secondary Structure Elements of Bacterial Effectors

**Figure 5:**
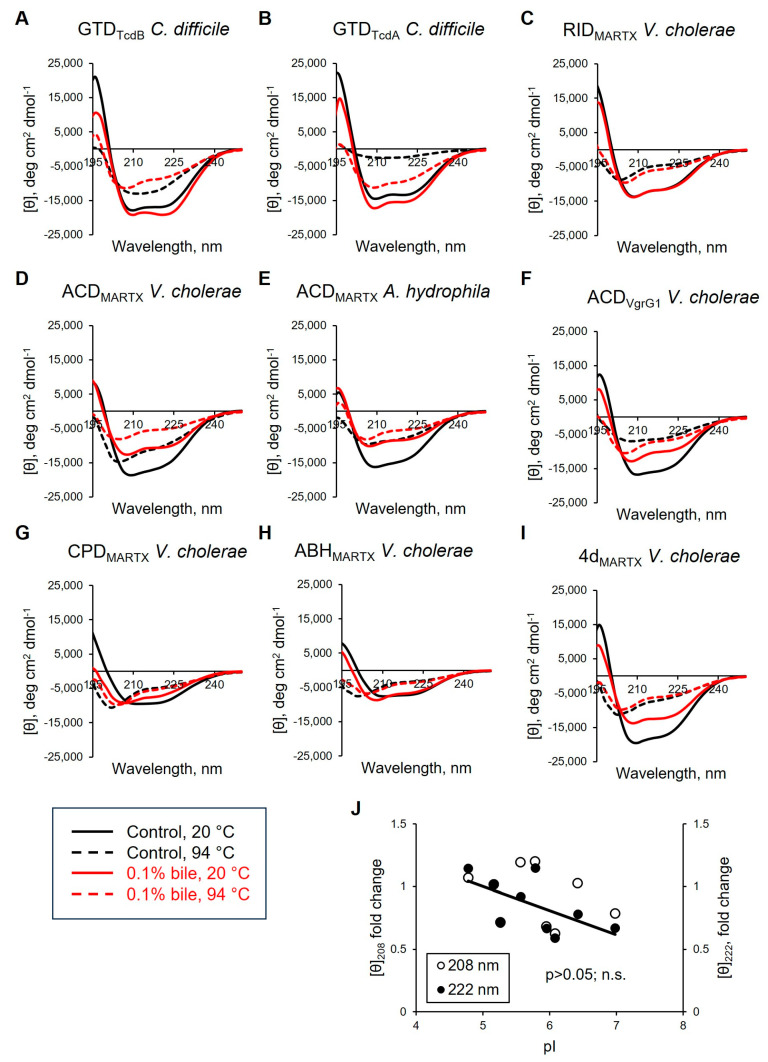
Bile disrupts the secondary structure of bacterial effectors. (**A**–**I**) Far-UV CD spectra (194 nm–250 nm) of bacterial effectors at 20 °C (solid lines) and 94 °C (dashed lines) in the presence (red) and absence (black) of 0.1% bile *w*/*v*. (**J**) Fold change in ellipticity (θ) at 208 or 222 nm of the bile-treated protein over the untreated control was plotted against the isoelectric point (pI) of each tested effector, and data were fit with a linear equation. *p*-values were determined from correlation analysis; n.s., not significant. See also Figures S4–S6.

To better understand the effects of bile on the structural stability of the effectors and avoid a potential bias of the interference with chaotropic agents, we utilized far-UV circular dichroism (CD) spectroscopy to measure changes in the secondary structure of selected effector domains upon their thermal unfolding (Figure 5; Figures S4–S6). In general, thermal denaturation of proteins is accompanied by flattening of the maxima and minima peak characteristics for α-helices (a peak at 190 nm and deeps at 208 and 222 nm) and β-sheets (a peak at 195 and a deep at 218 nm), and forming a prominent minima peak at ~200 nm characteristic of random coil. The reconstruction of the effectors’ secondary structure composition in the absence of bile by BeStSel software (Version 1.3.230210) [52] revealed that the α-helical and parallel β-strand content drops sharply upon thermal denaturation, reflecting unfolding, albeit not a complete loss, of these elements (Figure S5). Of all the secondary structure elements, only the antiparallel β-strand content is notably increased in a reciprocal manner in the same range of temperatures. This increase likely reflects the formation of new intermolecular bonds upon protein precipitation. It can be noticed that the melting temperatures for these proteins previously established by differential scanning fluorimetry [11] approximately coincided with the inception of the above transitions (Figure S5; green dashed lines), while melting points detected in the present study via near-UV CD measurements, characteristic for melting of tertiary structure elements, i.e., global unfolding [60], mostly matched the half-transition points for the secondary element profiles (Figure S5; blue dashed lines).

The effects of bile on the effectors’ CD spectra (Figure 5) can be separated into three distinct groups. In the first group, the GTD of TcdA and TcdB and, particularly, RID_MARTX_ were remarkably resistant to bile as judged by minor perturbations of their CD profiles at 20 °C (Figure 5A–C). Moreover, at intermediate and high temperatures, bile even stabilized the secondary structure elements of GTD_TcdA_ and RID_MARTX_ as reflected in the smaller changes in the characteristic 208/222 nm deeps, overall smoother melting profiles, and a lack of the sharp melting transitions of the predicted secondary structure elements (Figures S4–S6). This resistance to bile correlated with the low susceptibility of these effectors to collisional quenching (Figure 2B,E) and minor effects of bile on their chymotrypsinolysis (Figure 3B,D). In this group, only GTD_TcdB_ measurably responded to bile in collisional quenching and limited proteolysis experiments (Figure 2A and Figure 3A).

The second group, comprising three ACD constructs from different toxins, showed prominent signs of destabilization by bile in CD spectra (Figure 5D–F) and were sensitized by bile to collisional quenching (Figure 2F,G,K) and cleavage by chymotrypsin (Figure 3E,F,K).

Whereas the first two groups showed a strong correlation between the three sets of experiments, such correlation was not obvious for the third group, which comprised *V. cholerae* CPD_MARTX_ (*A. hydrophila* CPD_MARTX_ was not tested by CD) and *A. hydrophila* ABH_MARTX_. These proteins showed only minor changes in CD spectra, regardless of whether they responded to bile strongly (as CPD, Figure 2I and Figure 3H) or weakly (as ABH; Figure 3I) in other experiments. ABH does not have Trp residues and could not be tested for collisional quenching. We attribute this poor correlation to the substantial presence of β-elements in the secondary structure of these proteins (PDBID: 6IMP and 3EEB) [61,62] that are both destabilized (parallel β-sheets; Figures S5 and S6) and formed (antiparallel β-sheets) upon protein unfolding and subsequent precipitation, accompanied by the formation of intermolecular bonds, respectively.

At 94 °C, most effectors showed similar CD profiles with and without bile, as is anticipated for profoundly unfolded proteins. Yet, in some cases, bile either favored retaining partial secondary structure (Figure 5B,F) or promoted unfolding at high temperature (Figure 5D). Such a discrepancy, although puzzling, may reflect the amphipathic nature of bile that both promotes protein aggregation (thus, excluding it from analysis at high temperature (see below)) or, on the contrary, works as an emulsifier, keeping hydrophobic substances suspended in the solution. The latter observation was evident from the greater transparency of many samples in the presence of bile upon visual inspection after the analysis. The exact outcome, therefore, is specific to the particular protein and is difficult to predict.

### 3.6. Bile Potentiates Precipitation of Effector Proteins

**Figure 6:**
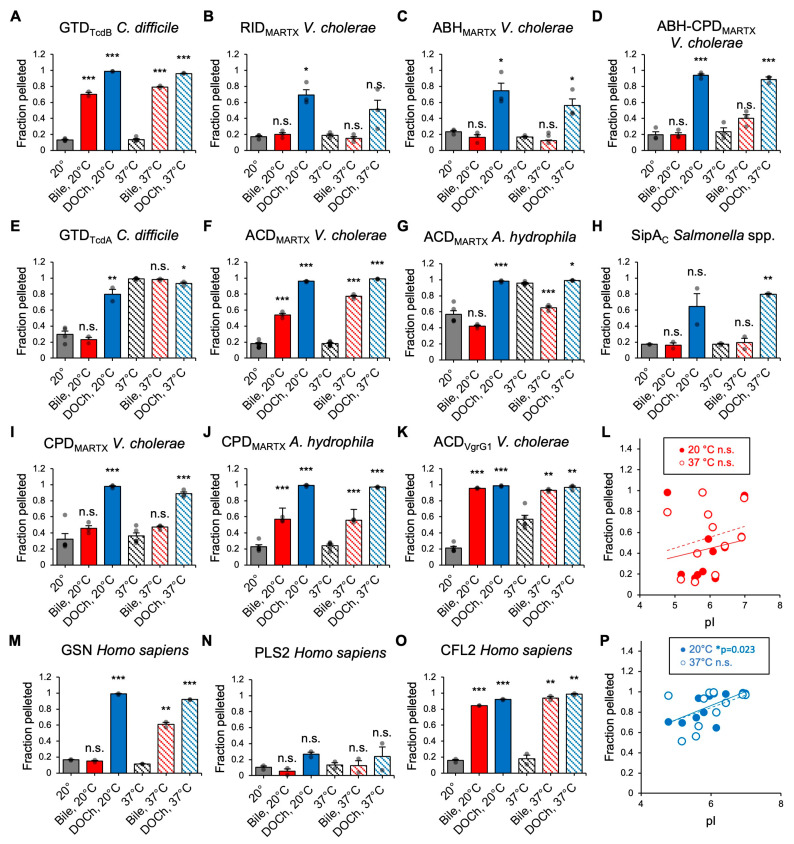
Bile and bile salts instigate precipitation of bacterial effector domains. (**A**–**K**,**M**–**O**) High-speed centrifugation assays were performed on bacterial effectors (**A**–**K**) and mammalian actin-binding proteins (**M**–**O**) at 20 (solid) and 37 °C (striped) in the presence of 0.25% bile *w*/*v* (red) or 0.25% DOCh *w*/*v* (blue). Individual data points are shown as grey dots. Data from at least three experiments are shown as mean ± SE. *p*-values were determined using two-tailed Student’s *t*-test (as compared to the respective untreated control); *, *p* < 0.05; **, *p* < 0.01; ***, *p* < 0.001; n.s., non-significant. (**L**,**P**) Fractions pelleted of each tested protein at 20 (solid linear fit line) and 37 °C (dashed linear fit line) in the presence of bile (**L**) or DOCh (**P**) were plotted against the proteins’ isoelectric points (pI). *p*-values were determined from correlation analysis.

Reduced solubility of proteins due to exposed hydrophobic residues can be used as an indirect reporter of protein denaturation if it is measured far from the protein’s isoelectric point (pI). Therefore, we assessed the effects of bile and DOCh on the solubility of bacterial toxins from *V. cholerae*, *A. hydrophila*, *C. difficile*, and *S. typhimurium* by pelleting upon ultracentrifugation at 20 and 37 °C (Figure 6).

We observed that DOCh promoted precipitation and pelleting of all tested bacterial effectors under both temperatures (Figure 6A–K). In contrast, precipitation of only selected toxins was promoted by bile (Figure 6A,F,J,K), while the solubility of others (e.g., RID, ABH, and CPD of *V. cholerae* MARTX toxin) was not affected (Figure 6B,C,I). Furthermore, bile increased the solubility of ACD_MARTX_ of *A. hydrophila*, the least thermostable of all the tested domains [11], which precipitated to a significant degree on its own at both tested temperatures (Figure 6G). Interestingly, while the ACD_VgrG1_ of T6SS *V. cholerae* toxin VgrG1 also self-precipitated to a substantial degree at 37 °C, its solubility was compromised by bile (Figure 6K). Similarly, the solubility of the ACD_MARTX_ of *V. cholerae* was reduced by bile, while the domain was soluble on its own at both temperatures (Figure 6F). Since the three ACD effectors share 60–68% identity [63] and overall structural organization, the observed differences indicate that the effects of bile can be dictated by relatively minor differences in structure and thermodynamic stability of the target proteins. Interestingly, the effect of bile on the solubility of the GTD of TcdA and TcdB also differed (Figure 6A,E), mirroring their response to bile in collisional quenching experiments (Figure 2A,E). GTD_TcdA_ was soluble at 20 °C and nearly completely precipitated at 37 °C, and its solubility was neither improved nor aggravated by bile at either temperature (Figure 6E). In contrast, despite being soluble at both temperatures, GTD_TcdB_ was notably precipitated by both bile and DOCh (Figure 6A). SipA_C_ was soluble at both temperatures, and its solubility was moderately compromised by DOCh, but not by bile (Figure 6H).

The mixed effects of whole bile and bile acids on different effectors suggest complex, context-dependent processes behind the observed destabilization, in agreement with previous observations on non-toxic proteins [59,64]. Accordingly, mammalian reference proteins CFL2, PLS2, and GSN, responded variably to the presence of whole bile and DOCh (Figure 6M–O). DOCh potentiated the precipitation of GSN at both temperatures, while bile increased the precipitation only at 37 °C (Figure 6M). Both bile and DOCh promoted precipitation of CFL2 but not PLS2 (Figure 6N,O), which correlated with their comparative susceptibility to collisional quenching (Figure 2L,M).

Bile-instigated toxin aggregation was also detected as the appearance of smeared high molecular weight species on native PAGE and dimerized species on non-reduced SDS-PAGE (Figure S7), further illustrating the precipitating effects of bile and DOCh.

### 3.7. Bile Inhibits the Specific Activity of CPD by Promoting Oxidation of the Catalytic Cysteine

**Figure 7:**
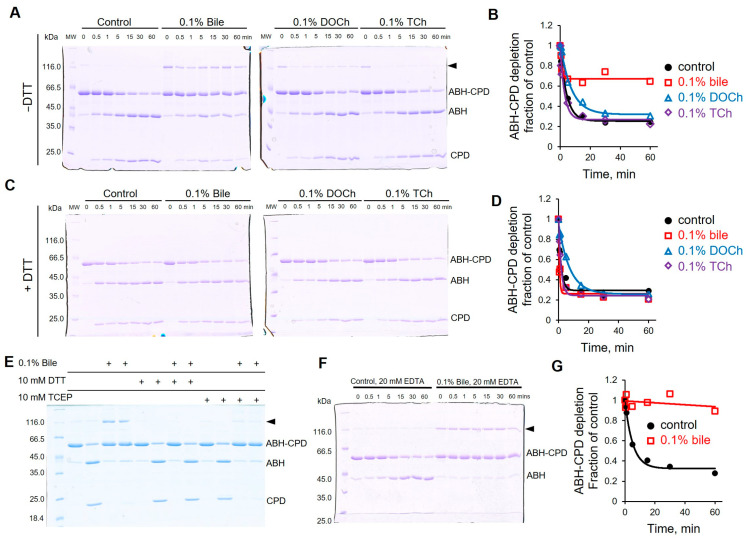
Bile reversibly inhibits cleavage of ABH-CPD_MARTX_ in a concentration-dependent manner and instigates dimer formation independent of transition metals. (**A**–**D**) Experiments were conducted in the absence of reducing agent (**A**,**B**) or in the presence of 10 mM DTT (**C**,**D**) for the indicated period of time. Representative SDS-PAGE images of ABH-CPD cleavage in the presence of 0.1% *w*/*v* bile, DOCh, or TCh (**A**,**C**) were quantified by densitometry (**B**,**D**). (**E**) SDS-PAGE image of ABH-CPD cleavage in the presence of bile and either 10 mM DTT or 10 mM TCEP. For each condition, samples were incubated for 0 and 15 min, as indicated. (**F**,**G**) A representative SDS-PAGE image of ABH-CPD cleavage for the indicated time intervals in the absence and presence of 0.1% *w*/*v* bile and 20 mM EDTA (**F**) was quantified by densitometry (**G**). Appearance of a dimer is indicated by an arrowhead in (**A**,**E**,**F**). See also Figures S8 and S9.

Since bile distorts the structure of effector proteins, we speculated that their functional activities might also be affected. Cysteine protease domain (CPD) is found in numerous bacterial toxins, including MARTX, where it proteolytically releases respective effector domains to the cytosol of the host cell, upon activation by inositol-hexakisphosphate (IP_6_) [61,65,66]. Using a two-domain construct ABH-CPD_MARTX_ containing a native CPD cleavage site between the domains as a model, we observed prominent inhibition of the CPD activity in the presence of bile (Figure 7A,B and Figure S8A). Notably, the bile treatment was accompanied by the appearance of a protein band twice the molecular weight of the original ABH-CPD construct when run on SDS-PAGE under non-reducing conditions (Figure 7A). The disappearance of this extra band under reducing conditions confirms its origin as a disulfide bond-connected ABH-CPD_MARTX_ dimer (Figure 7C). Since the only cysteine residue in CPD_MARTX_ is the catalytic cysteine of the active site, its engagement in the disulfide bond converts the enzyme into a catalytically inactive state. Less expectedly, ~70% of monomeric ABH-CPD_MARTX_ was also fully protected from cleavage in the presence of bile (Figure 7A,B). Deoxycholate (DOCh) and taurocholate (TCh) also favored the formation of the dimer, albeit in lesser quantities. However, they imposed no (by TCh) or weak (by DOCh) inhibition of the proteolytic cleavage of the monomeric species (Figure 7A,B). The addition of dithiothreitol (DTT), as a reducing agent in the course of the cleavage reaction, released the bile-imposed cleavage inhibition of ABH-CPD_MARTX_ monomer (Figure 7C,D and Figure S8B), tentatively suggesting that oxidation of the catalytic cysteine, different from disulfide bond formation, may be involved. This supposition was confirmed by probing the availability of intact cysteine by a thiol-specific tetramethyl rhodamine-5 maleimide (TMR-5-maleimide) (Figure S9). To avoid a possible interference between the probe and bile, bile was removed by passing the sample through a size-exclusion gravity column before labeling (Figure S9A). More effective incorporation of TMR-5-maleimide into ABH-CPD_MARTX_ in the absence of bile (Figure S9D) supported the hypothesis that the specific activity of the CPD protease is inhibited due to bile-induced oxidation of the catalytic cysteine. Further analysis using DCP-Rho1, a sulfenic acid targeting fluorescent rhodamine probe, following bile treatment and gel filtration indicated no significant difference in the sulfenic acid content of control and bile-treated samples (Figure S9E).

Surprisingly, tris(2-carboxyethyl)phosphine (TCEP), another potent reducing agent, acting as a direct donor of electrons, failed to rescue the CPD_MARTX_ activity in the presence of bile, while effectively preventing the dimer formation (Figure 7E). Since neither DOCh nor TCh caused the inhibition comparable to that of bile, we speculated that the trace presence of transition metals (e.g., Fe^3+^), known to mediate physiological redox reactions, may have caused the observed oxidation. Yet, the addition of 20 mM ethylenediaminetetraacetic acid (EDTA) had no effect on the inhibition or the formation of the dimer (Figure 7F), arguing against the role of transition metals in the inhibition of CPD by bile.

To validate that the bile-induced inhibition is not limited to the ABH-CPD_MARTX_ construct, we repeated the cleavage using a MARTX construct containing four effector domains, 4d_MARTX_, and observed overall comparable DTT-sensitive inhibition by bile (Figure S10). However, the additional Cys residue located in RID_MARTX_ resulted in the formation of more complex oligomers, which complicated analysis (Figure S10). ACD_VgrG1_ *V. cholerae*, also having a single cysteine residue, forms a dimer in the presence of bile in a similar manner (Figure S11), suggesting bile’s broad cysteine oxidation ability.

### 3.8. Bile Inhibits the Activities of V. cholerae Effectors ACD and VopF

**Figure 8:**
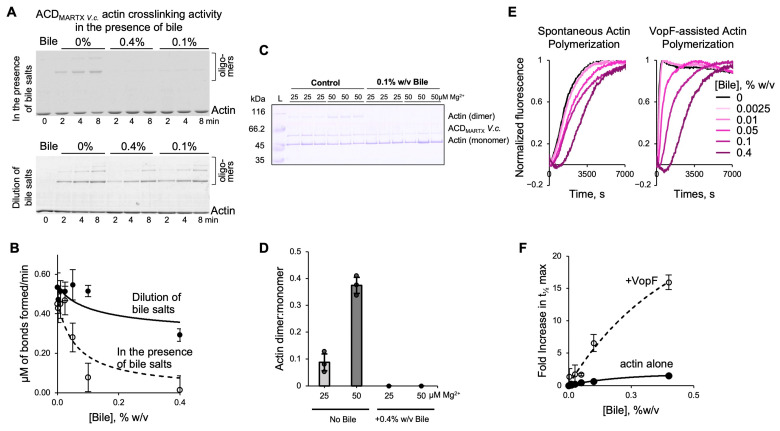
Bile inhibits specific activities of ACD and VopF. (**A**–**D**) Rates of actin crosslinking by ACD in the absence and presence of bile (**A**, **top**) or after bile dilution (the original concentrations are indicated) (**A**, **bottom**) were analyzed by SDS-PAGE (**A**,**B**) and quantified by densitometry (**C**). Data from 3 independent experiments are shown as mean ± SE. Ratio of actin monomer to dimer during actin crosslinking assays in the presence of 0.4% *w*/*v* bile was analyzed by SDS-PAGE (**C**) and quantified by densitometry (**D**). (**E**,**F**) Effects of bile on actin polymerization in bulk in the absence (spontaneous) and presence of VopF (VopF-assisted) were assessed as time to half maximum (t_1/2max_) of pyrene fluorescence intensity (**E**) and plotted versus bile concentration (**F**). Data from 3 independent experiments are shown as mean ± SD.

As part of the MARTX toxin, ACD_MARTX_ is secreted into the intestinal lumen and, therefore, is likely to be exposed to bile under physiological conditions. We found that the ability of ACD_MARTX_ to covalently crosslink actin monomers into oligomers [38,54,67,68,69] was notably reduced in the presence of bile (Figure 8A,B). The inhibition of ACD_MARTX_ was reversible, as dilution of the bile/effector mixture to sub-inhibitory concentrations resulted in substantial, albeit not complete, recovery of the crosslinking activity (Figure 8A,B). The inhibition of undiluted ACD was confirmed under suboptimal crosslinking conditions (i.e., low Mg^2+^ [54,68]), necessary to achieve temporary resolution at high doses of the enzyme (Figure 8C,D).

To further explore whether the observed inhibition is applicable to non-enzymatic toxins, we evaluated the effects of bile on VopF, an actin pointed-end processive polymerase and nucleator [40,70]. Using the bulk pyrenyl-actin polymerization assays, we found that as bile concentration increases, a slowdown in actin polymerization was observed, recorded as an increased time to half maximal (t_1/2max_; Figure 8E,F). While bile also inhibited the spontaneous polymerization of actin (Figure 8E,F), the effects on VopF-mediated polymerization were notably more dramatic, supporting bile’s ability to negatively affect diverse activities of various bacterial toxins.

## 4. Discussion

Antibacterial properties of bile are not understood fully but are recognized to be mediated by the disruption of bacterial membranes, induction of RNA and DNA damage, promotion of oxidative stress, and precipitation of intracellular proteins [18,19]. These effects represent a multifactorial challenge for bacterial cells and are remediated by activation and overexpression of molecular chaperones (e.g., Hsp33, DnaK, and GroESL) [18,19]. Surprisingly, the effects of bile on proteinaceous toxins, particularly secreted toxins, are even less investigated and known mainly for the reversible inhibition of *C. difficile* TcdA/B toxins [19,36,71,72]. This gap in knowledge is unfortunate as proteinaceous toxins and their effector domains are the primary factors of morbidity and mortality associated with bacterial infections, enabling bacterial pathogens to evade immune protection and colonize host organisms. In this work, we attempted to address this deficiency by characterizing the effects of bile on a representative group of model effector domains from *V. cholerae*, *A. hydrophila*, * C. difficile*, and *S. enterica*. While many toxins are delivered into the host cells via injection upon direct contact between a microbe and its host (e.g., via T3SS, T4SS, T6SS delivery machinery), others are secreted to the host’s fluids. While effector domains of such secreted toxins were the primary focus of our analysis, we also tested domains of toxins secreted by other systems, which is justified by the need to test for potential selectivity and by the ability of bile to affect proteins in the cytoplasm of bacterial cells [18,19].

In this study, we report that bile indeed neutralizes (and facilitates neutralization by other factors, e.g., digestive proteases) a broad range of bacterial toxins and effector domains. This effect is achieved via several mechanisms that include (1) promoting surface exposure of hydrophobic residues, (2) destabilizing their secondary structure elements, (3) causing protein aggregation, (4) promoting the oxidation of cysteine residues, and (5) enabling more effective proteolysis by chymotrypsin. Together, the effects are translated to a reduced specific activity as exemplified by model enzymatic and structural effectors, CPD, ACD, and VopF. Note that complete protein denaturation is neither achieved under the tested conditions nor necessary to cause the inactivation, as long as the effectors are shifted sufficiently from their native state to perturb their function. Furthermore, the observed cysteine oxidation resulted in the formation of disulfide bonds, which should interfere with host cell entry by toxins secreted to the extracellular space (e.g., by T1SS and T2SS). Likely due to a combination of the above effects (apart from proteolysis), bile effectively protects cultured enterocytes from *A. hydrophila* secreted toxins aerolysin, hemolysins, and *RtxA* (Figure 1; Table S1).

Notice that the above effects can be deduced to a stronger binding of bile components to the unfolded protein state, which, according to the principles of linked equilibrium, promotes unfolding. This is not surprising given that a bulky hydrophobic part of bile acids is expected to interact with the hydrophobic side chains of the proteins, inaccessible in the folded state but exposed in the denatured state. Accordingly, bile acids have been shown to structurally destabilize *C. difficile* TcdA and TcdB toxins, inhibiting their binding to the receptor and neutralizing cytotoxicity [36,72,73,74]. On a similar note but with a strikingly different outcome, DOCh promotes the transition of anthrax toxin from the pre-pore to pore form, which is structurally less compact and more unfolded, but functionally more active [75]. In this case, the pathogen takes advantage of the promoted partial unfolding that favors the pathogenesis, just as the unfolding of anthrax Lethal Factor (LF) and Edema Factor (EF) under the influence of acidic pH in the early endosome favors the pathogenesis by promoting their translocation to the host cell cytosol [76].

While bile acid effects on a random protein are likely to be destabilizing, some proteins have evolved to be stabilized by bile, or to transition into a state of altered activity, if this state preferentially interacts with bile components. Such transitions agree with the role of bile as an environmental cue and have indeed been demonstrated for several bacterial proteins. Thus, binding of bile acids to the partially unfolded lipocalin(-like) fold of *Vibrio* and other bacterial spp. T3SS2-component system drives the equilibrium towards a more stable, folded state, triggering downstream transcription of bacterial toxin effectors [21,27,77,78]. As part of this mechanism, the bile-provoked changes in the protein structure favor further stabilization of these bacterial bile-sensing transcription systems via phosphorylation and disulfide bond formation [25,79]. Given the small size of bile acids and their compositional complexity, the interaction of several different molecules of bile acids with a single molecule of protein is expected, potentially leading to an even more complex blend of stabilizing and destabilizing effects, complicating their examination.

Accordingly, we found that bile did not uniformly affect all the toxin effectors and varied both in strength and type of the predominant changes, reflecting a complex character of the bile-protein interaction. In past studies, this complexity has prevented establishing a correlation between protein properties and their susceptibility to bile effects [19]. This complexity can be exemplified by the effects on protein precipitation: bile can both favor the precipitation of a protein by denaturing it, but also promote solubility by shielding exposed hydrophobic surfaces, working as a biological emulsifier (Figure 6; compare the promoted precipitation of ACD_MARTX_ *V. cholerae* and improved solubilization of otherwise more vulnerable ACD_MARTX_ *A. hydrophila*). Since these effects may be dominated by local or global influences, reflecting differences in protein nature, the final outcome is defined by a balance between these influences, precluding establishing links between basic effector characteristics (e.g., pI, aliphatic index, melting temperature, etc.) and precipitation (Figure 6; Table S2). For similar reasons, the bile effects observed by different methodologies correlate well but do not perfectly converge, demonstrating various degrees of susceptibility of various target proteins. This is not entirely surprising given that each method detects different reporters, contributing to the complexity of the observed effects.

Despite this complexity, our approach to assessing multiple characteristics of a large group of effector domains has proved successful, as it has allowed important connections not observed previously to be established. Specifically, the exposure of Trp residues to solution quenchers showed a linear correlation with the isoelectric points (pI) of the effectors (Figure 2O), with more acidic proteins (carrying more net negative charge) being less affected by bile. This observation is logical as most bile acids are also charged negatively at neutral pH and, therefore, should be repulsed, preventing their effective binding to the acidic effectors. The most notable exception, SipA_C_, only confirmed the rule while demonstrating the important role of local effects, i.e., charged residue distribution in proximity of the only Trp residue (Figure S2I). The correlation could still be detected in CD experiments, showing that the effectors more prone to surface exposure of the hydrophobic core in the presence of bile (e.g., all ACD effectors) also demonstrated larger drops in the secondary structure content, particularly α-helices and parallel β-sheets (Figure 5 and Figure S5). Surprisingly, chemical denaturation by GdmCl confirmed the exposure of Trp residues by bile but revealed no major effects of bile on the stability (Figure 4), likely pointing to cancellation of the bile denaturation effects due to shielding from a more potent and highly ionic denaturant.

The negative correlation between effectors’ acidity and neutralization by bile points to pressure on bacterial effectors to evolve towards lower pI values. It is reasonable to ask, then, why have all the tested effectors not evolved towards higher acidity? The answer to this apparent paradox may be in the presence of another potent broad-range immune protector, α-defensins. α-Defensins, which in humans are represented by neutrophil HNP1-3 defensins and intestinal HD5/HD6, are innate immune peptides that are capable of neutralizing a remarkably broad range of secreted bacterial toxins [1,2,3,7,80,81] by acting upon the toxins’ conformational plasticity and low thermodynamic stability [4,8]. As opposed to bile, α-defensins are highly cationic and, as such, should be more active against acidic effectors, accounting for the described paradox.

Despite this difference, it did not escape our attention that bile acids and defensins share some common properties. Thus, the amphipathic nature and rigid structure are among the properties of defensins that enable toxin inactivation [8] and are also characteristics of bile. Amphipathicity bestows reasonable solubility in water and enables interactions with both polar and non-polar protein compartments. A rigid structure (provided by either a rigid *cyclophenanthrene* nucleus or disulfide bonds) may be essential for “cutting” into the protein structure, leading to its distortion. Despite ~10-fold difference in size (~0.39 kDa and ~3.3 kDa for bile acids and defensins, respectively), both are small compared to the average size of affected proteins. On the other hand, α-defensins are more effective at much lower concentrations (typically slight molar access over the concentrations of affected proteins), and cause notably less damage to host proteins than bile [4,82].

While sharing the abilities to expose Trp residues and promote precipitation and proteolytic cleavage, the effects of α-defensins are notably more selective towards proteinaceous toxins, causing little to no detrimental effects against tested human proteins [4]. In contrast, bile did not discriminate between bacterial and tested human proteins, namely GSN, CFL2, and PLS2 (Figure 2, Figure 3 and Figure 6). GSN was precipitated by bile and DOCh at physiological temperatures (Figure 6M). CFL2 was heavily precipitated and showed higher Trp quenching in the presence of bile and DOCh (Figure 2M and Figure 6O). PLS2 was the most resistant to the effects of bile (Figure 2L, Figure 3M and Figure 6N), which correlated with its lower pI (pI of 5.2), as compared to more basic CFL2 and GSN (pI of 7.66 and 5.90, respectively).

Interestingly, one can predict that cationic defensins and anionic bile acids may be electrostatically attracted, and such an association is likely to be inhibitory. Whether such a possibility indeed takes place in vivo and whether it can be alleviated (e.g., by differential compartmentalization of these agents) under physiological conditions remains to be established. It will be important and interesting to test these hypotheses in future studies.

At the functional level, both bile and α-defensins notably inhibit the activity of model enzymatic effectors, ACD and CPD, but accomplish that by different mechanisms. Thus, HNP1 inhibits the overall proteolytic activity of CPD but also favors extensive cleavage in unnatural locations within the adjacent MARTX domains by exposing the hydrophobic sites otherwise hidden in the folded protein [4]. In the presence of bile, CPD cleaves the ABH-CPD_MARTX_ construct at a single site, just as the intact CPD, but only a fraction of the construct remains enzymatically active. We identified one reason for this pattern of inhibition, potentiated oxidation of the catalytic residues leading to the formation of the disulfide bond stabilized dimer, but it is also likely oxidation that does not involve the disulfide bond formation. The latter supposition is supported by the release of the inhibition in the presence of DTT (Figure 7A–D) and discriminative labeling of cysteines in bile-treated and untreated constructs by TMR-5-maleimide (Figure S9D). Decompensated disulfide stress (akin to that caused by hypochlorite (OCl^−^)) was proposed to be a mechanism for bile-induced protein precipitation inside bacterial cells, presumably due to the inhibition of proteins involved in the production of NADPH and/or the maintenance of GSH/GSSG homeostasis [18,19]. Notice, however, that these mechanisms cannot explain the increased oxidation under the in vitro conditions of our experiments, suggesting that bile can impose oxidative stress by different means. Two obvious factors that can account for the observed oxidation are (1) the exposure of cysteine residues from the protein core to the surface and (2) precipitation, bringing protein surfaces in immediate proximity, favoring the bond formation. While important, these factors may not account for the entirety of the effects, as they do not explain the inhibition of CPD activity in the monomeric ABH-CPD_MARTX_ construct. Since EDTA did not release the inhibition, other bile components than transition metals may favor cysteine oxidation via unknown mechanisms. With CPD being a conserved toxin effector found across many bacterial strains, its inhibition by bile presents a broad mechanism of toxin neutralization.

The disulfide bond formation has consequences far beyond the inhibition of the catalysis by Cys-containing effectors. There is strong evolutionary pressure against cysteines in secreted toxins, such that *V. cholerae* and *V. vulnificus* MARTX toxins contain only 2 and 3 cysteine residues, all catalytic, per ~4500 and ~5200 total residues, respectively. Similarly, lethal and edema factors of *Bacillus anthracis* contain one and no cysteines per ~800 residues, respectively. This corresponds to ~0.05% of cysteine content on average in these toxins, as compared to 2–2.5% in cytoplasmic and 3–3.5% in extracellular non-toxin proteins. The reason behind this pressure is the translocation of the effectors as unfolded chains via a narrow pore that was demonstrated for the anthrax toxin [9] and implied for MARTX [11]. Since disulfide bonds preclude the open conformation, factors promoting their formation (e.g., bile) will inevitably block the effector entry and prevent toxicity. Whereas this mechanism does not apply to *Vibrio* VopF and *Salmonella* SipA toxins that are secreted directly to the host cell cytosol via the T3SS and would not be in contact with bile in the extracellular space, these effectors may be among the proteins that aggregate and precipitate inside the bacterial cell, in the presence of bile [19]. While structural destabilization by bile is sufficient to abolish the enzymatic activity of at least some effectors (Figure 7), bacterial toxin effectors containing Cys residues, such as CPD_MARTX,_ would be particularly vulnerable to structural destabilization, exposure of hydrophobic regions (e.g., binding pockets), and subsequent oxidation.

It is important to pinpoint the limitations of this study that should be addressed in the future. First, we used isolated effector domains rather than full-length toxins. It is conceivable that even the domains that function in the cytoplasm of host cells as independent entities (e.g., domains of MARTX toxins), protect each other from the effects of immune factors (e.g., bile and defensins) upon secretion and before the domains are separated by CPD-mediated cleavage. Indeed, our own data on the lower susceptibility of CPD’s Trp residues to collisional quenching when it is fused with the ABH domain (Figure 2D,I) favor such a possibility. Next, we explored the effects of a single dose of bile and only a single bile acid (DOCh) in most of the experiments, out of many that can be present in various physiological doses. Stronger effects of bile than DOCh on several of the tested effector domains (Figure 2) suggest that other bile components may be more active than DOCh and are likely to exert at least some level of selectivity to specific targets.

## 5. Conclusions

To summarize, this study introduces bile as a potent immune factor capable of neutralizing a broad range of bacterial effectors, possibly comparable in breadth of action to α-defensins. Structural and functional experiments detailed in this study indicate that bile interacts with bacterial toxins by hydrophobic and ionic interactions, favoring more positively charged toxin effectors. The amphipathic properties of bile acids enable interactions with hydrophobic surfaces, shifting the equilibrium toward unfolding. Secondary structure destabilization in the presence of bile promotes precipitation and increases the likelihood of cysteine oxidation as buried hydrophobic regions become surface exposed. Virulence regulation mediated by pathogens’ conserved bile sensors [77] likely includes mechanisms enabling the surmounting of this ability [73]. It is tempting to speculate that successful infections in the small intestine would depend on the ability of pathogenic bacteria to overcome the antimicrobial effects of bile, but also on the expression of sufficient amounts of toxin to persist against inactivation by bile. Both enhanced expression of virulence factors/toxins (to compensate for the bile-caused inactivation) and alleviated expression (to avoid wasting valuable resources if the neutralization is highly successful) can be anticipated depending on a particular context.

## Data Availability

Data is contained within the article or Supplementary Materials.

## Supplementary Materials

The following supporting information can be downloaded at https://www.mdpi.com/article/10.3390/biom15111539/s1. Figure S1. Bile prevents membrane damage of IEC-18 cells caused by *A. hydrophila* supernatant; Figure S2. Solved or AlphaFold2-predicted structures of the tested proteins of interest; Figure S3. Bile does not inhibit chymotrypsin proteolytic activity; Figure S4. The addition of bile disrupts the secondary structure of bacterial toxin effectors; Figure S5. Secondary structure analysis of MARTX effector domains; Figure S6. Secondary structure analysis of GTD_TcdA_, GTD_TcdB_, and ACD_VgrG1_; Figure S7. Addition of bile promotes aggregation and oxidation of MARTX bacterial effector; Figure S8. Bile reversibly inhibits the CPD activity; Figure S9. CPD activity is inhibited by thiol oxidation in the presence of bile; Figure S10. Bile reversibly inhibits cleavage of 4d_MARTX_ and instigates Cys oxidation; Figure S11. *V. cholerae* ACD_VgrG1_ is oxidized in the presence of 0.1% *w*/*v* bile; Table S1. LC-MS/MS identified secreted, membrane-damaging toxins in *A. hydrophila* supernatant after overnight growth; Table S2. Summary table of all protein parameters for the studied bacterial effectors.

## Abbreviations

The following abbreviations are used in this manuscript:

ABH	α/β-hydrolase domain
ACD	Actin crosslinking domain
β-ME	β-mercaptoethanol
CD	Circular dichroism
CFL2	Cofillin 2
CID	Collision-induced dissociation
CPD	Cysteine protease domain
DCP	Diethyl chlorophosphite
DMEM	Dulbecco’s modified Eagle medium
DOCh	Deoxycholate
DTT	Dithiothreitol
EF	Edema factor
EDTA	Ethylenediaminetetraacetic acid
FBS	Fetal bovine serum
GdmCl	Guanidinium hydrochloride
GSH	Reduced glutathione
GSN	Gelsolin
GSSG	Oxidized glutathione
GTD	Glycosyltransferase domain
HD5	Human defensin 5
HD6	Human defensin 6
HNP1	Human neutrophil peptide 1
HNP2	Human neutrophil peptide 2
HNP3	Human neutrophil peptide 3
IAA	Iodoacetamide
IP_6_	Inositol-hexakisphosphate
K_SV_	Stern–Volmer coefficient
LatB	Latrunculin B
LB	Luria–Bertani medium
LF	Lethal factor
LPS	Lipopolysaccharide
MARTX	Multifunctional autoprocessing repeats-in-toxin
NADPH	Nicotinamide adenine dinucleotide phosphate
NEM	N-ethylmaleimide
PBS	Phosphate-buffered saline
pI	Isoelectric point
PA	Protective antigen
PLS2	Plastin 2
PMSF	Phenylmethylsulfonyl fluoride
RID	Rho-inactivation domain
SDS	Sodium dodecyl sulfate
SipA_C_	SipA C-terminal domain
T1SS	Type I secretion system
T2SS	Type II secretion system
T3SS	Type III secretion system
T4SS	Type IV secretion system
T6SS	Type VI secretion system
TCEP	Tris(2-carboxyethyl)phosphine
TCh	Taurocholate
TEAB	Triethylammonium bicarbonate
TMR	Tetramethylrhodamine
