# Supplementary material for: Effects of Bile on Pathogenic Vibrio, Aeromonas, and Clostridioides spp. Toxin Effector Domains
Source: Biomolecules. 2025 Nov 1;15(11):1539. doi: 10.3390/biom15111539 (PMC12649987; doi:10.3390/biom15111539)
Supplement: Supplementary file 1 [file biomolecules-15-01539-s001.zip › Supplementary Information.pdf]

Supplementary Material for

Effects of Bile on Pathogenic *Vibrio*, *Aeromonas*, and *Clostridioides* spp. Toxin Effector Domains

Jaylen E. Taylor, David Heisler, Eshan Choudhary, Elena Kudryashova, Dmitri S. Kudryashov

This file includes:

Supplementary Figures (S1-S11)

Supplementary Tables (S1 and S2)

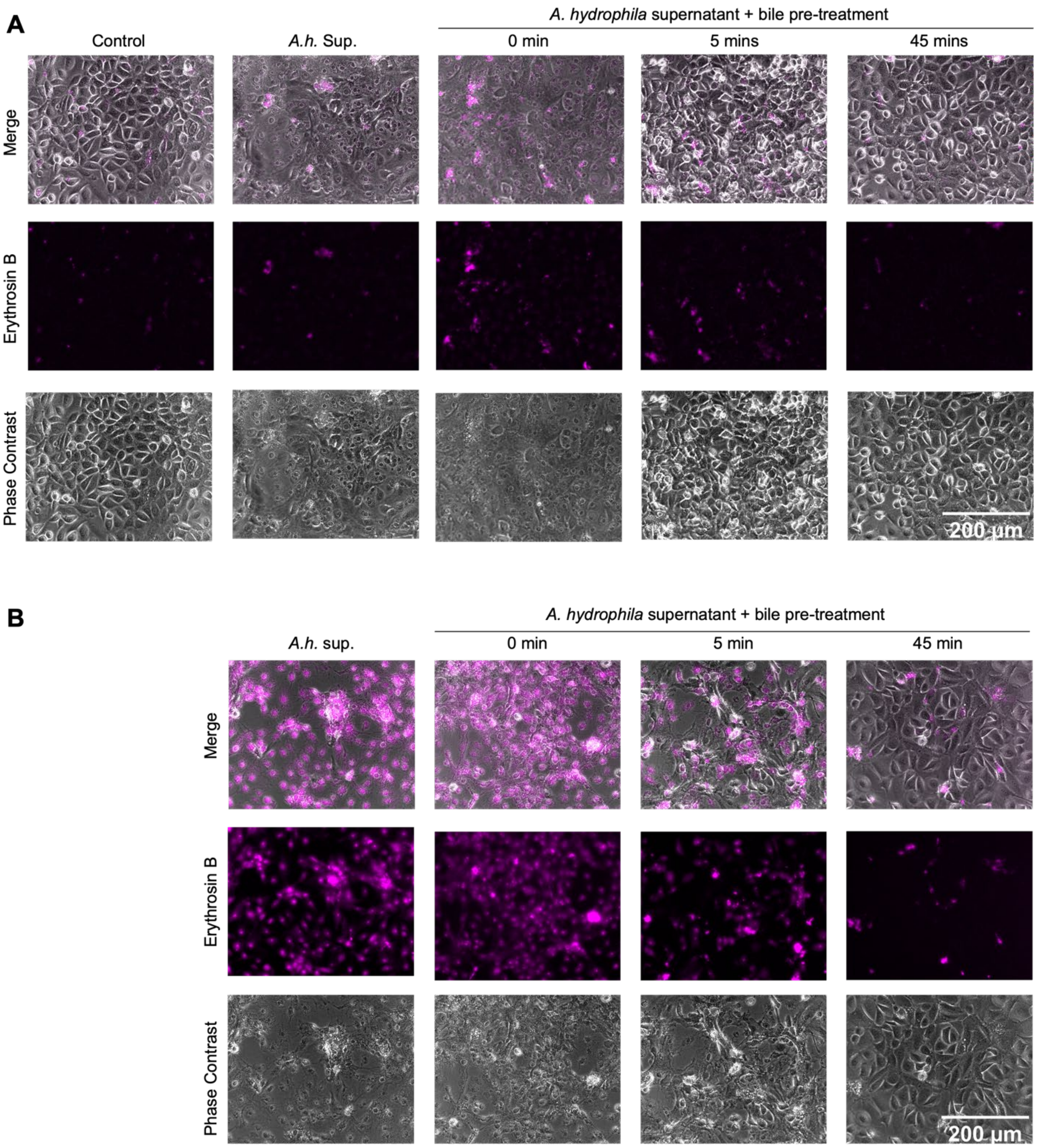

Figure S1. Bile prevents membrane damage of IEC-18 cells caused by *A. hydrophila* supernatant. (**A,B**) Micrographs show phase contrast and erythrosin B fluorescence images of IEC-18 cells treated with *A. hydrophila* supernatant, untreated or pre-treated with bile for 1 h (A) or 5 h (B). Related to Figure 1.

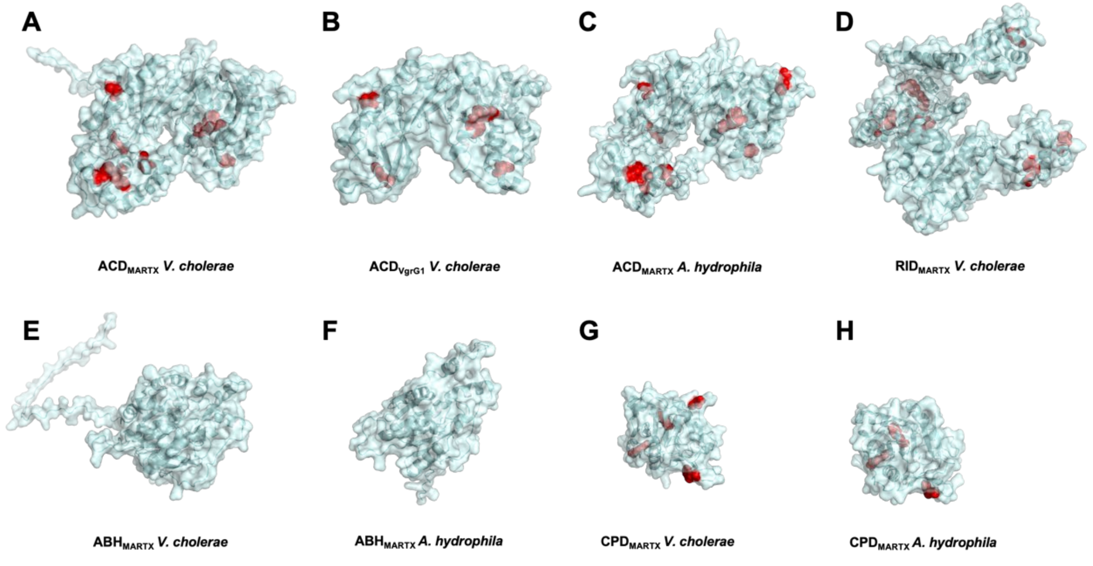

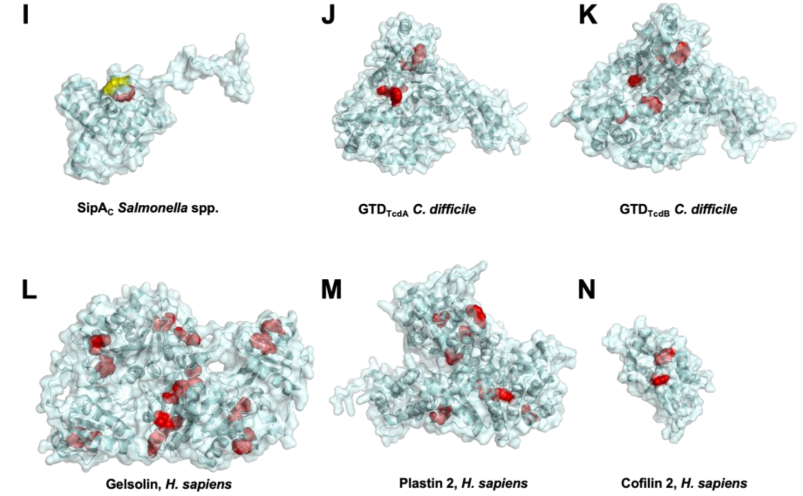

O

Protein Name	Organism	#TRP	PDB
**ACD$_{MARTX}$**	*V. cholerae*	8	AlphaFold2
**ACD$_{VgrG1}$**	*V. cholerae*	5	4dth
**ACD$_{MARTX}$**	*A. hydrophila*	9	AlphaFold2
**RID$_{MARTX}$**	*V. cholerae*	10	AlphaFold2
**ABH$_{MARTX}$**	*V. cholerae*	0	AlphaFold2
**ABH$_{MARTX}$**	*A. hydrophila*	0	AlphaFold2
**CPD$_{MARTX}$**	*V. cholerae*	4 (*3)	3eeb
**CPD$_{MARTX}$**	*A. hydrophila*	3	AlphaFold2
**SipA$^{425-684}$**	*S.e. typhi*	1	8c4e
**GTD$_{TcdA}$**	*C. difficile*	5	4r04
**GTD$_{TcdB}$**	*C. difficile*	5	7lou
**ADF/Cofilin-2**	*H. sapiens*	2	7m0g
**Gelsolin**	*H. sapiens*	15	6jeg
**Plastin-2**	*H. sapiens*		AlphaFold2

Figure S2. Solved or AlphaFold2-predicted structures of the tested proteins of interest. (**A-N**) The structure images were created using PyMOL. Trp residues are labeled in red; Arg657 of SipA$_{C}$ (I) is labeled in yellow. (**O**) The number of tryptophan residues in each protein and a corresponding PDB ID (where applicable) are listed. Related to Figure 2.

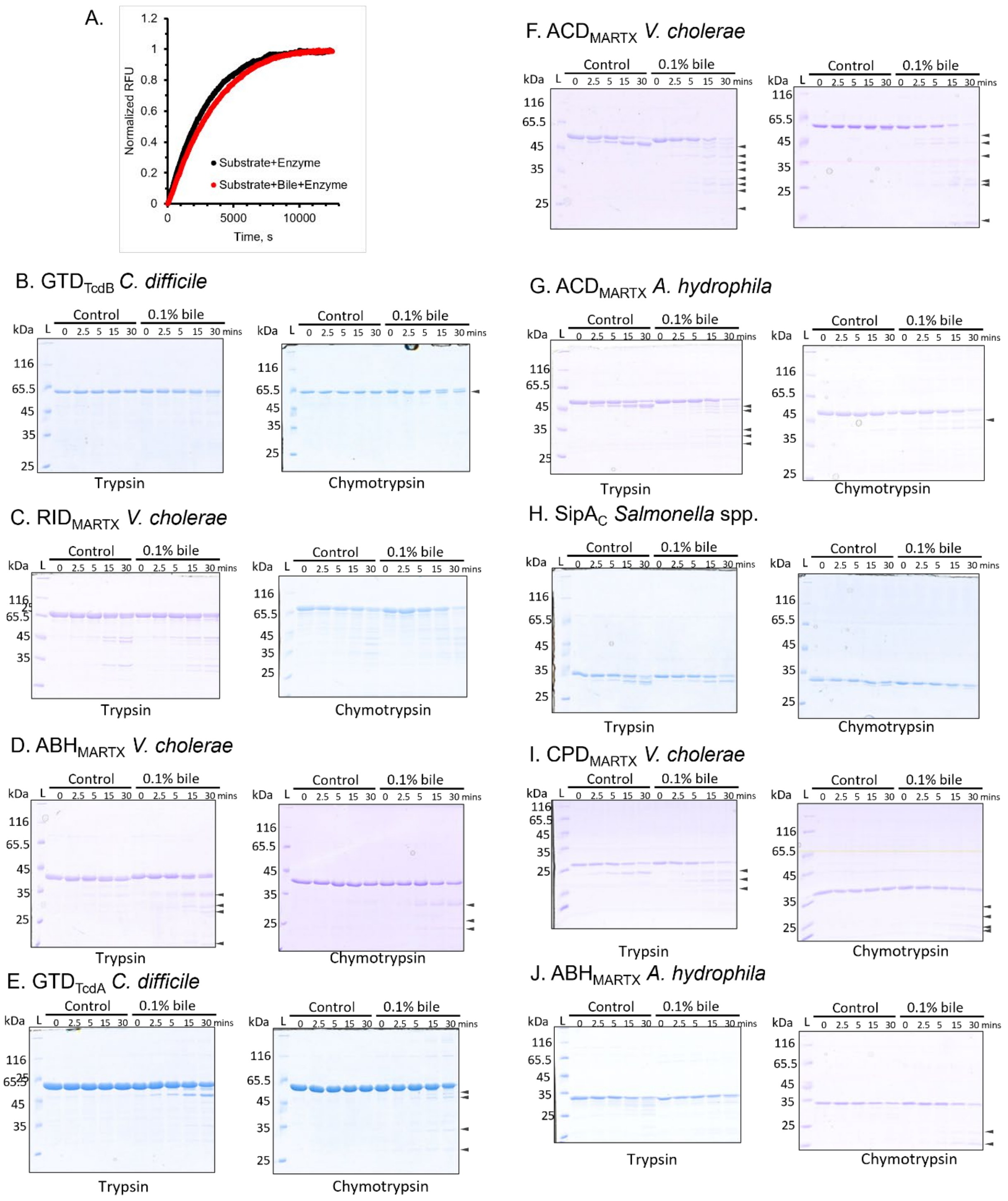
A.
Normalized RFU
Time, s
Substrate+Enzyme
Substrate+Bile+Enzyme
B. GTD$_{TcdB}$ *C. difficile*
C. RID$_{MARTX}$ *V. cholerae*
D. ABH$_{MARTX}$ *V. cholerae*
E. GTD$_{TcdA}$ *C. difficile*
F. ACD$_{MARTX}$ *V. cholerae*
G. ACD$_{MARTX}$ *A. hydrophila*
H. SipA$_{C}$ *Salmonella* spp.
I. CPD$_{MARTX}$ *V. cholerae*
J. ABH$_{MARTX}$ *A. hydrophila*
Control
0.1% bile
kDa
L
0 2.5 5 15 30 0 2.5 5 15 30 mins
116
65.5
45
35
25
Trypsin
Chymotrypsin

Figure S3 continued.

K. CPD_{MARTX} *A. hydrophila*

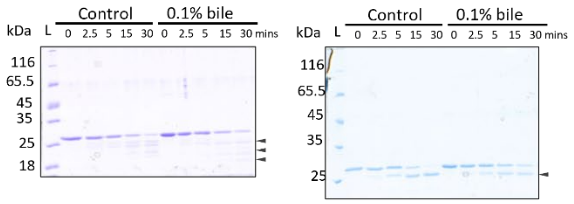

L. ACD_{VgrG1} *V. cholerae*

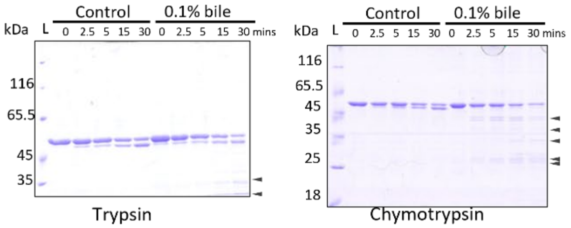

M. CFL2, *Homo sapiens*

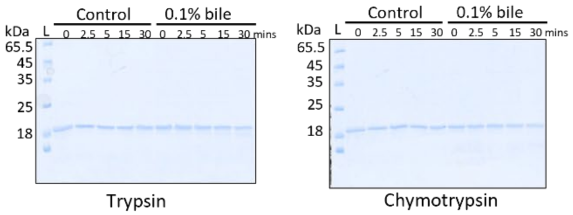

N. PLS2, *Homo sapiens*

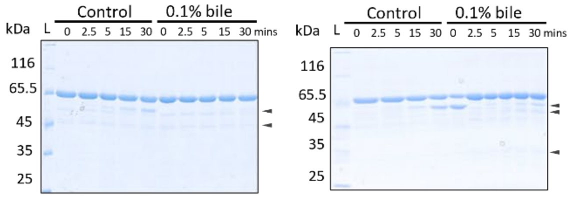

O. GSN, *Homo sapiens*

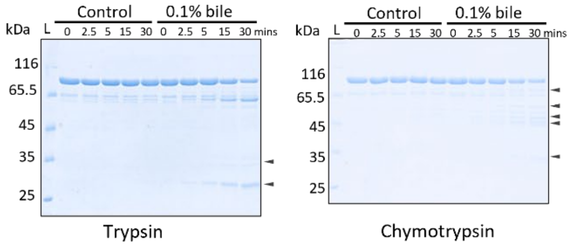

Figure S3. Bile does not inhibit chymotrypsin proteolytic activity. (A) Fluorogenic chymotrypsin substrate (5 µM) was cleaved by chymotrypsin (1:2057 molar ratio of the substrate to the enzyme). Fluorescence of cleaved substrate was monitored at an excitation of 380 nm and emission of 460 nm in the presence and absence of 0.1% bile. (B-O) Representative gels from trypsin (left) and chymotrypsin (right) limited proteolysis assay quantified in figure 3A-N. The positions of proteolytic products that appear only in the presence of bile are indicated by arrowheads. Related to Figure 3.

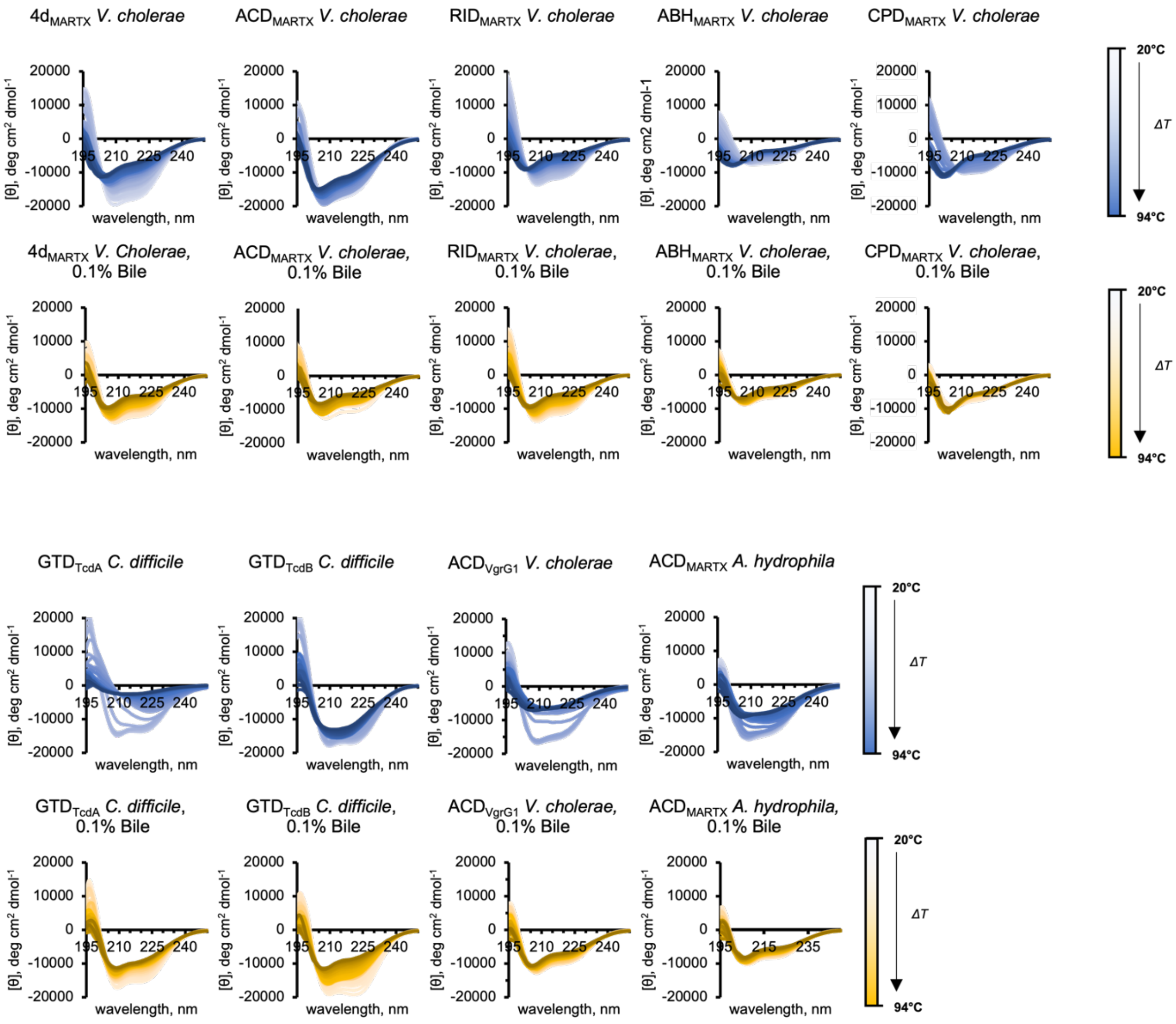

Figure S4. The addition of bile disrupts the secondary structure of bacterial toxin effectors. Far-UV CD spectra (194 nm – 250 nm) of 0.25 mg/mL of bacterial toxin effectors were measured every 2 °C from 20 °C to 94 °C (color gradient) at a rate of 2 °C per min in the presence (yellow) and absence (blue) of 0.1% bile w/v. Related to Figure 5.

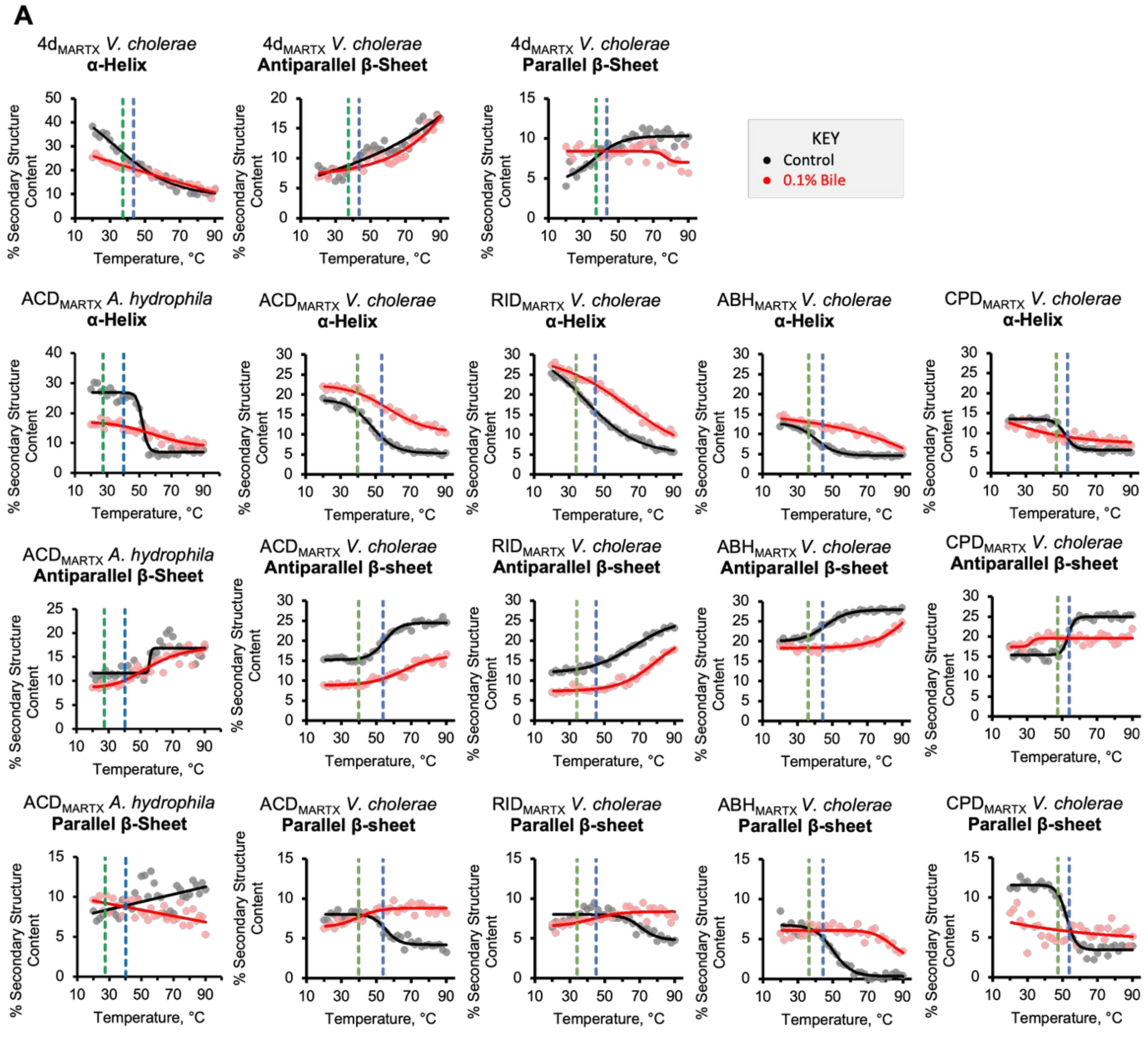

B

MARTX Effector Domain Melting Temperature (Tm), °C

METHOD		4d *V.c.*	ACD *V.c*	ACD *A.h.*	RID *V.c.*	ABH *V.c.*	CPD *V.c.*
Far-UV Circular Dichroism	Secondary Structure	43.4	53.6	40.2	45.1	44.3	53.8
Differential Scanning Fluorimetry (DSF)	Tertiary Structure	37.3	39.7	27.4	34.2	36.3	47.7

Kudryashova *et al.* 2014, Molecular Microbiology, 92, 1056 - 1071

Figure S5. Secondary structure analysis of MARTX effector domains. (**A**) Far-UV CD spectra of the bacterial effectors in the presence (red) and absence (black) of 0.1% bile obtained every 2 °C from 20 °C to 94 °C were analyzed using BestSel to predict α-helical, antiparallel β-sheet, and parallel β-sheet secondary structure content. Secondary structure content was plotted against temperature. (**B**) Melting temperatures for secondary and tertiary structures for each effector construct determined previously [Kudryashova *et al., 2014,* Molecular Microbiology, 92, 1056-1071] are presented as tabular data in (B) and shown as blue (for secondary structure T_m) and green (for tertiary structure T_m) dotted lines in (A). Related to Figure 5.

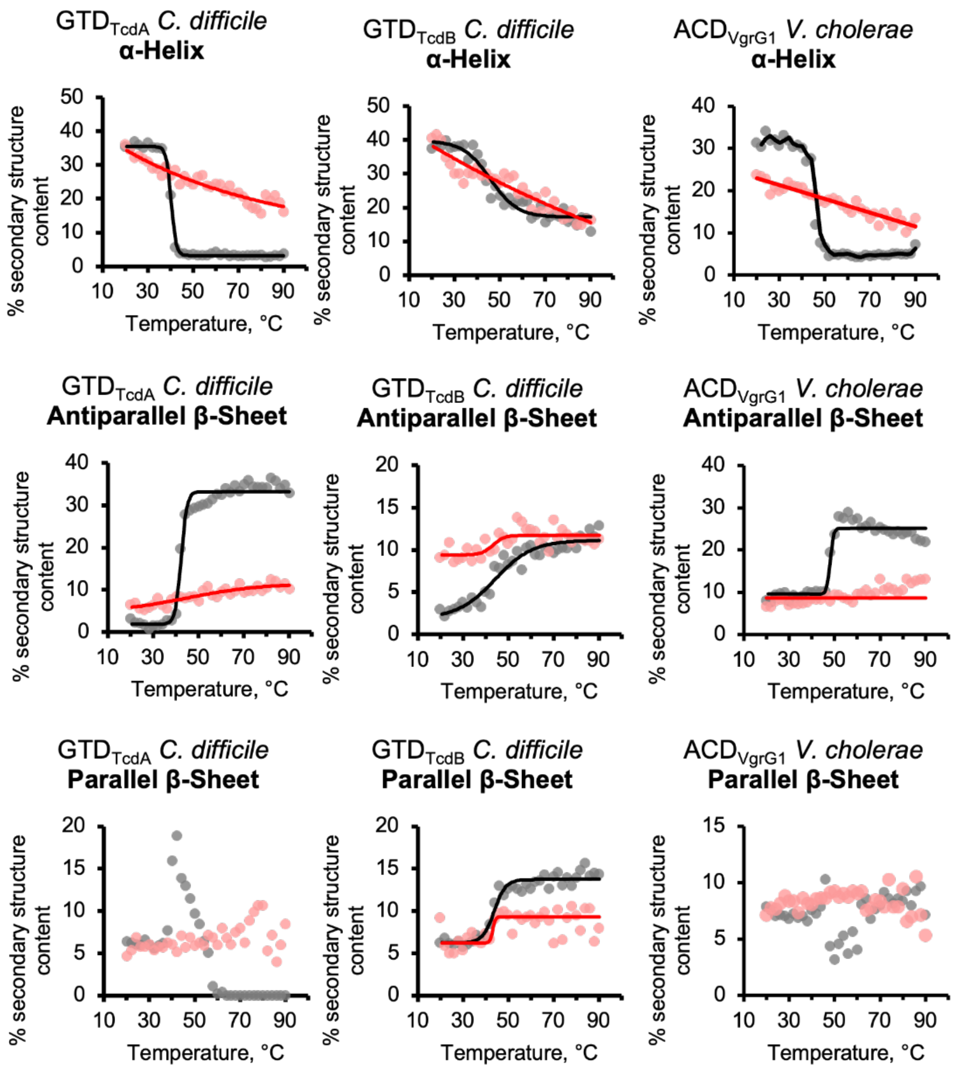

Figure S6. Secondary structure analysis of GTD$_{TcdA}$, GTD$_{TcdB}$, and ACD$_{VgrG1}$. Far-UV CD spectra of the bacterial effectors in the presence (red) and absence (black) of 0.1% bile obtained every 2 °C from 20 °C to 94 °C were analyzed using BestSel to predict α-helical, antiparallel β-sheet, and parallel β-sheet secondary structure content. Secondary structure content was plotted against temperature. Related to Figure 5.

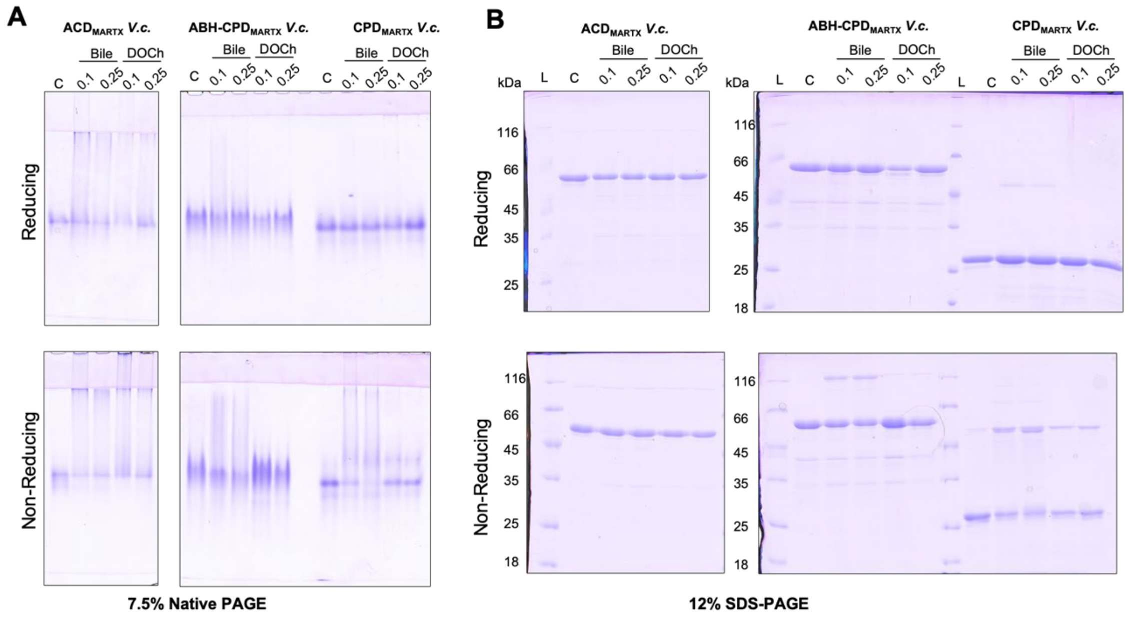

Figure S7. Addition of bile promotes aggregation and oxidation of MARTX bacterial effector. MARTX effector domains (5 μM) were resolved on native PAGE (A) and SDS-PAGE (B) after incubation in the absence and presence of the indicated amounts of bile or DOCh for 30 min at 37 °C. Related to Figure 6.

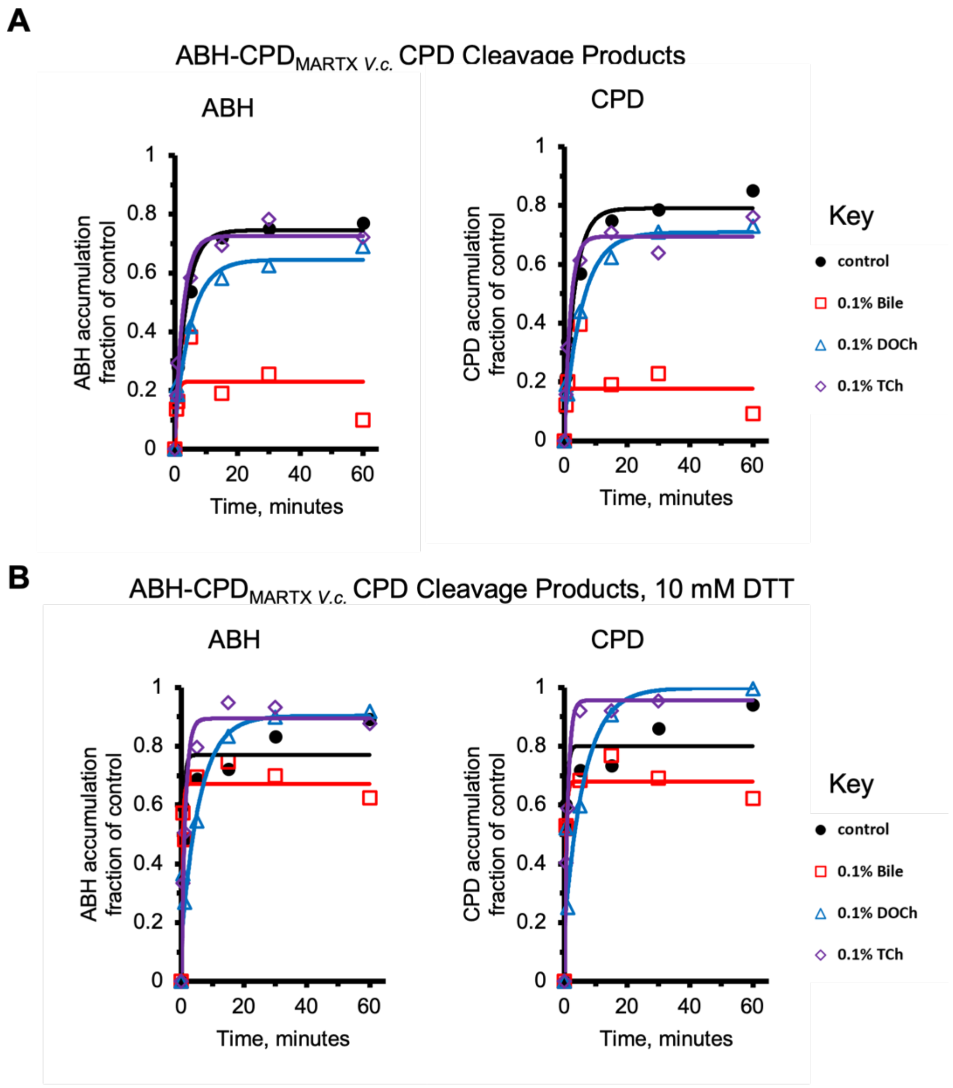

Figure S8. Bile reversibly inhibits the CPD activity. Densitometry analysis of the Coomassie-stained SDS-PAGE gel images shown on Fig. 7A,C. Activity of CPD *in cis* was assessed as described in the Methods section. Experiments were conducted in the absence of reducing agent (A) or in the presence of 10 mM DTT (B). Related to Figure 7.

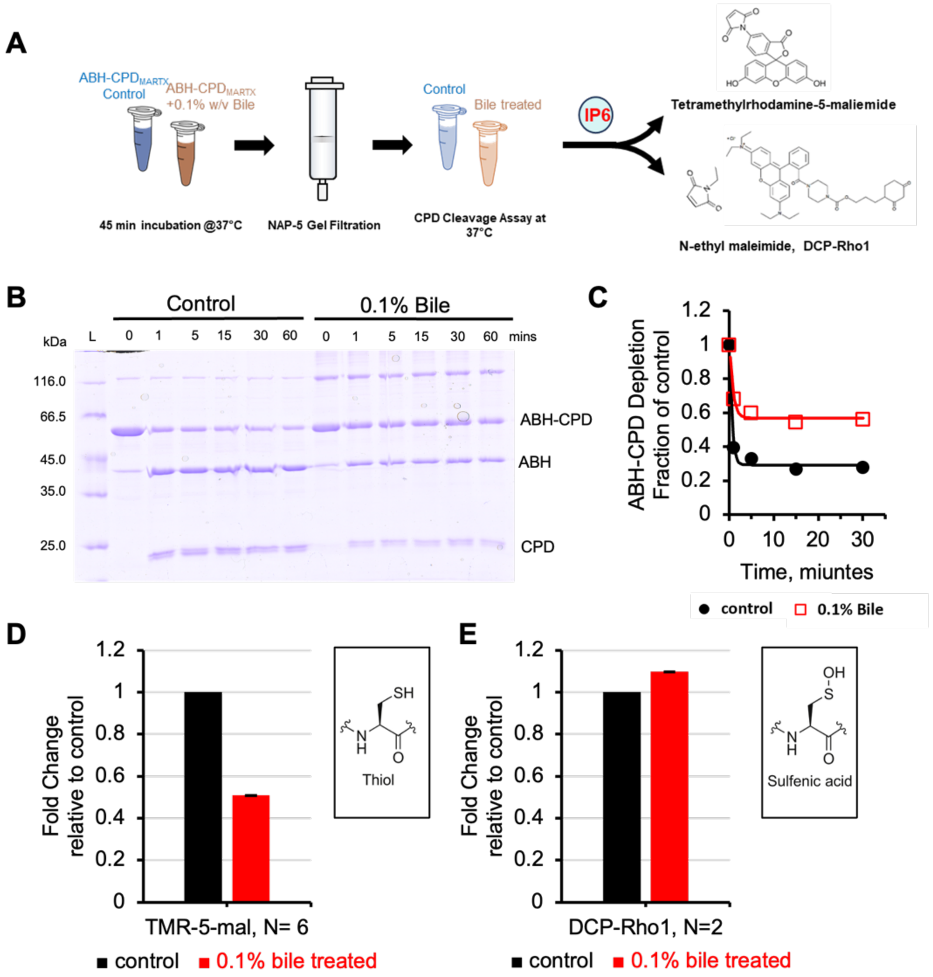

Figure S9. CPD activity is inhibited by thiol oxidation in the presence of bile. (**A**) Experimental workflow: ABH-CPD was incubated in the presence and absence of 0.1% w/v bile for 45 mins at 37°C. MARTX construct was then passed through NAP-5 gel filtration column to remove "free" bile components. Post-gel filtration, CPD activity was activated by adding 50 μM IP_6 and quenched with 1 mM NEM. Following the cleavage, the samples were labeled with TMR-5-maleimide or DCP-Rho1. (**B**) The samples at each indicated time point were resolved on non-reducing SDS-PAGE 12% gel. (**C-E**) The fraction of ABH-CPD cleaved (C), fold change of TMR-5-maleimide-labeled protein (D), and fold change of DCP-Rho1 labeled protein (E) relative to the untreated control were quantified. Related to Figure 7.

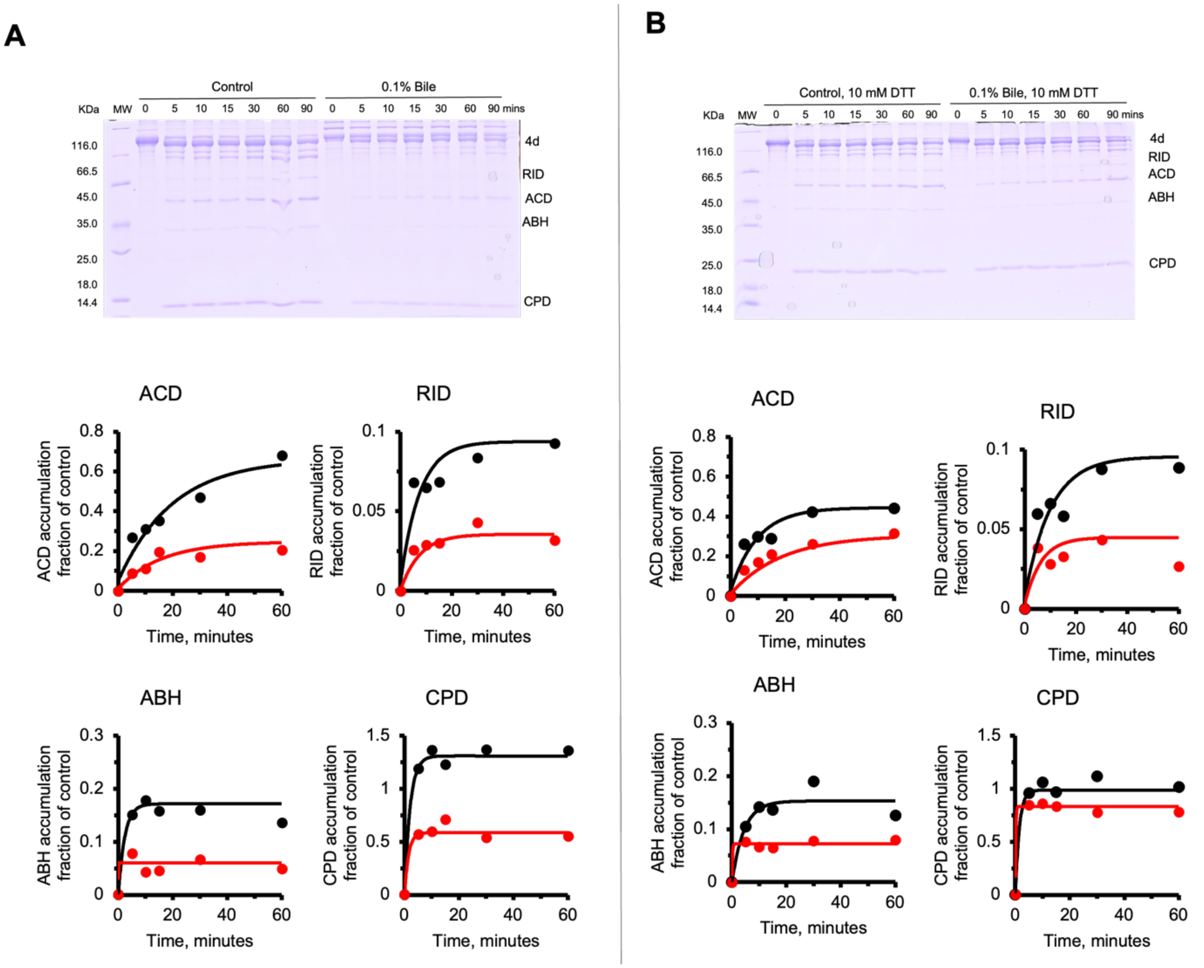

Figure S10. Bile reversibly inhibits cleavage of $4d_{MARTX}$ and instigates Cys oxidation. Activity of CPD *in cis* in the context of $4d_{MARTX}$ construct was assessed as described in the Methods section. (**A,B**) Experiments were conducted in the absence of reducing agent (A) or in the presence of 10 mM DTT (B) for the indicated period of time. SDS-PAGE images of $4d_{MARTX}$ cleavage in the presence of 0.1 % w/v bile, DOCh, or TCh were quantified by densitometry (plots). Related to Figure 7.

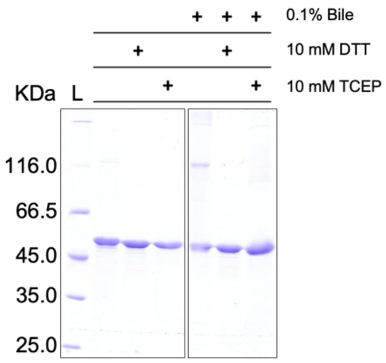

Figure S11. *V. cholerae* ACD$_{VgrG1}$ is oxidized in the presence of 0.1% w/v bile. 5 µM ACD$_{VgrG1}$ from *V. cholerae* in 20 mM TRIS-HCl, 150 mM NaCl, pH 7.5 was incubated at 4 °C overnight followed by a room-temperature incubation (25 °C) for an hour in the presence and absence of 0.1% w/v bile supplemented with 10 mM DTT or 10 mM TCEP. Samples were visualized by non-reducing SDS-PAGE. Related to Figure 8.

Locus	Protein	Unique Spectral Count	Coverage
AHA_0438	Aerolysin, *AerA*	352	38.54
AHA_1359	MARTX, *RtxA*	20	3.56
AHA_1512	Hemolysin, *Ahh1*	684	71.66
AHA_2697	Hypothetical protein	6	3.99
AHA_2809	TlyC, putative Mg^{2+}/Co^{2+} transporter	1	2.96

Table S1. **LC-MS/MS identified secreted, membrane-damaging toxins in *Aeromonas hydrophila* supernatant after overnight growth.** Related to Figure 1.

					Collisional Quenching				
					K_{SV}			K_{SV} FC	
Bacterial Strain	Toxin	Effector(s)	pI	AI	Control	Bile	DOCh	Bile	DOCh
C. difficile	TcdB	GTD	4.78	84.4	1.76	2.71	2.15	1.54	1.22
V. cholera	MARTX	RID	5.17	86.78	3.48	2.94	3.84	0.84	1.10
V. cholera	MARTX	3d	5.21	80.67					
V. cholera	MARTX	4d	5.26	80.27	2.36	2.85	3.50	1.21	1.48
V. cholera	MARTX	ABH	5.57	75.64					
V. cholera	MARTX	ABH-CPD	5.64	76.23	1.02	1.17	1.35	1.15	1.32
C. difficile	TcdA	GTD	5.79	93.76	1.75	1.54	2.43	0.88	1.39
V. cholera	MARTX	ACD	5.95	73.27	1.32	3.08	3.17	2.33	2.40
A. hydrophila	MARTX	ACD	6.08	79.11	2.09	3.31	3.48	1.58	1.67
S. enterica	SipA	Cterm	6.15	75.44	1.22	3.34	2.74	2.74	2.25
V. cholera	MARTX	CPD	6.42	74.41	1.42	2.30	1.20	1.62	0.85
A. hydrophila	MARTX	ABH	6.42	77.91					
A. hydrophila	MARTX	CPD	6.9	78.25	2.55	4.74	2.15	1.86	0.84
V. cholera	VgrG1	ACD	6.98	71.66	1.12	2.71	2.90	2.42	2.59

					GdmHCl Denaturation										
					EC_{50}			EC50, P-value	EC_{50} FC	Minimum average		Min., P-value	Maximum average		Max., P-value
Bacterial Strain	Toxin	Effector(s)	pI	AI	Control	Bile	Δ		Bile	Control	Bile		Control	Bile	
C. difficile	TcdB	GTD	4.78	84.4											
V. cholera	MARTX	RID	5.17	86.78	1.69	1.71	0.02	0.65	1.01	329.70	329.90	0.29	343.90	344.70	0.42
V. cholera	MARTX	3d	5.21	80.67											
V. cholera	MARTX	4d	5.26	80.27											
V. cholera	MARTX	ABH	5.57	75.64											
V. cholera	MARTX	ABH-CPD	5.64	76.23	1.22	1.37	0.15	0.03	1.12	323.70	329.10	0.01	341.70	342.90	0.03
C. difficile	TcdA	GTD	5.79	93.76											
V. cholera	MARTX	ACD	5.95	73.27	1.45	1.49	0.04	0.65	1.03	325.40	328.70	0.30	342.56	343.80	0.11
A. hydrophila	MARTX	ACD	6.08	79.11	1.84	1.82	-0.02	0.69	0.99	327.10	325.80	0.30	343.00	343.60	0.44
S. enterica	SipA	Cterm	6.15	75.44											
V. cholera	MARTX	CPD	6.42	74.41	1.15	1.20	0.05	0.23	1.04	324.80	330.90	0.00	342.60	344.70	0.15
A. hydrophila	MARTX	ABH	6.42	77.91											
A. hydrophila	MARTX	CPD	6.9	78.25	1.04	1.12	0.09	0.13	1.08	324.90	330.80	0.03	342.30	344.30	0.01
V. cholera	VgrG1	ACD	6.98	71.66	1.76	1.87	0.11	0.38	1.06	327.10	329.40	0.01	342.90	342.00	0.17

					Far-UV Circular Dichroism							
					Molar Ellipticity, θ				θ FC		Tm, °C	
Bacterial Strain	Toxin	Effector(s)	pI	AI	Control, 208 nm	Bile, 208 nm	Control, 222 nm	Bile, 222 nm	Bile, 208 nm	Bile, 222 nm	208 nm	222 nm
C. difficile	TcdB	GTD	4.78	84.4	-17447.21	-18655.47	-16733.84	-19156.04	1.07	1.14	42.1	41.8
V. cholera	MARTX	RID	5.17	86.78	-13635.32	-13852.63	-10960.78	-11212.20	1.02	1.02	39.8	44.4
V. cholera	MARTX	3d	5.21	80.67								
V. cholera	MARTX	4d	5.26	80.27	-19192.12	-13721.83	-16665.54	-11854.49	0.71	0.71	not calculated	not calculated
V. cholera	MARTX	ABH	5.57	75.64	-7265.53	-8669.21	-6937.08	-6391.62	1.19	0.92	46.2	40.6
V. cholera	MARTX	ABH-CPD	5.64	76.23								
C. difficile	TcdA	GTD	5.79	93.76	-14167.73	-16997.62	-13212.33	-15175.49	1.20	1.15	40.3	45.5
V. cholera	MARTX	ACD	5.95	73.27	-18532.08	-12649.90	-15673.27	-10454.17	0.68	0.67	50.9	47.7
A. hydrophila	MARTX	ACD	6.08	79.11	-16119.03	-10070.36	-13680.54	-8091.35	0.62	0.59	53.6	57.6
S. enterica	SipA	Cterm	6.15	75.44								
V. cholera	MARTX	CPD	6.42	74.41	-9029.59	-9279.80	-8716.13	-6822.12	1.03	0.78	55	50.8
A. hydrophila	MARTX	ABH	6.42	77.91								
A. hydrophila	MARTX	CPD	6.9	78.25								
V. cholera	VgrG1	ACD	6.98	71.66	-15744.24316	-12866.142	-14691.29671	-9850.256319	0.82	0.67	45.8	46.3

Table S2. Summary table of all protein parameters for the studied bacterial effectors. pI, isoelectric point; AI, aliphatic index; K_{SV}, Stern-Volmer coefficient; K_{SV} FC, Fold change in stern-Volmer coefficient (relative to control); EC_{50}, half maximal effective concentration; FC EC_{50}, fold change in half maximal effective concentration (relative to control); min., minimum wavelength; max, maximum wavelength; θ, molar ellipticity; θ FC, molar ellipticity fold change (relative to control); T_m, melting temperature.
